# Single-cell scattering and auto-fluorescence-based fast antibiotic susceptibility testing for gram-negative and gram-positive bacteria

**DOI:** 10.3389/fmicb.2023.1232250

**Published:** 2023-08-04

**Authors:** Sophie Dixneuf, Anne-Coline Chareire-Kleiberg, Pierre Mahé, Meriem El Azami, Chloé Kolytcheff, Samuel Bellais, Cyril Guyard, Christophe Védrine, Frédéric Mallard, Quentin Josso, Fabian Rol

**Affiliations:** ^1^BIOASTER Technology Research Institute, Lyon, France; ^2^bioMérieux SA, La Balme-les-Grottes, France; ^3^bioMérieux SA, Grenoble, France; ^4^bioMérieux SA, Marcy-l’Étoile, France; ^5^BIOASTER Technology Research Institute, Paris, France

**Keywords:** antibiotic susceptibility testing, growth-free, flow cytometry, label-free, *Escherichia coli*, *Staphylococcus epidermidis*, beta-lactam, aminoglycoside

## Abstract

In this study, we assess the scattering of light and auto-fluorescence from single bacterial cells to address the challenge of fast (<2 h), label-free phenotypic antimicrobial susceptibility testing (AST). Label-free flow cytometry is used for monitoring both the respiration-related auto-fluorescence in two different fluorescence channels corresponding to FAD and NADH, and the morphological and structural information contained in the light scattered by individual bacteria during incubation with or without antibiotic. Large multi-parameter data are analyzed using dimensionality reduction methods, based either on a combination of 2D binning and Principal Component Analysis, or with a one-class Support Vector Machine approach, with the objective to predict the Susceptible or Resistant phenotype of the strain. For the first time, both *Escherichia coli* (Gram-negative) and *Staphylococcus epidermidis* (Gram-positive) isolates were tested with a label-free approach, and, in the presence of two groups of bactericidal antibiotic molecules, aminoglycosides and beta-lactams. Our results support the feasibility of label-free AST in less than 2 h and suggest that single cell auto-fluorescence adds value to the Susceptible/Resistant phenotyping over single-cell scattering alone, in particular for the *mecA*+ *Staphylococcus* (i.e., resistant) strains treated with oxacillin.

## 1. Introduction

Developing new fast diagnostic tests is a critical part in the worldwide fight against antimicrobial resistance. Such tests could contribute to better pathogen-targeted therapeutic decisions as well as to lower costs of development of new drugs by facilitating clinical trials (Hughes, 2011). Over the last decade, various strategies aiming at shortening the time-to-result have been explored. The most promising ones rely on shortening or suppressing the bacterial culture step classically needed for biomass amplification. On the other hand, optical methods (i.e., microscopies, micro-spectroscopies, flow cytometry) have reached the sensitivity and specificity required for single-bacteria-cell-based diagnostic (Choi et al., 2014; Mulroney et al., 2017; Hong et al., 2018; Novelli-Rousseau et al., 2018; Li et al., 2019; Forsyth et al., 2021). This is particularly attractive in a flow cytometry mode, as a high diversity and complexity of cells can be analyzed based on multiple parameters and at a high pace, bringing rich and statistically relevant results. Several proposals have been made to discriminate susceptible and resistant bacteria strains using different labelling techniques in flow cytometry from early proof of concept (Walberg et al., 1996, 1997a,b) to clinical studies (Suller et al., 1997; Faria-Ramos et al., 2013; Huang et al., 2015; Silva et al., 2016), and recently to same-day confirmation of infection and antimicrobial susceptibility in a clinical setting (Mulroney et al., 2022). However, for all label-based trials, sample preparation is usually time-consuming and cumbersome. Fluorescent molecules used as labels can affect the pathogen and impact downstream analyses as those dyes are frequently cytotoxic. Fast optical diagnostic techniques featuring simplified and low-impact sample preparation are highly desired. In this context, Huang et al. extended their original multidimensional statistical analysis approach to a rapid (3 h), label-free-cytometry-based AST in blood culture by monitoring changes in the scattered light signals of the bacteria, thus focusing on morphological and structural changes induced by the antibiotic treatment on the bacterial cells rather than on chemical changes (Huang et al., 2018). Their demonstration focused on well-recognized Gram-negative (Gram-) multi-drug resistant species (*Escherichia coli*, *Klebsiella pneumoniae*, and *Acinetobacter nosocomialis*).

On the other hand, numerous endogenous molecules involved in the energy metabolism of live bacteria exhibit fluorescence at specific excitation/emission wavelengths, making them attractive as non-invasive probes for microbiological diagnostic and characterization (Ivnitski et al., 1999; Ammor, 2007). Coenzymes involved in the aerobic respiration of bacteria, more precisely in the tricarboxylic acid cycle (also known as Krebs cycle), exhibit absorption/emission maxima in the near-UV and blue regions (Krebs and Johnson, 1980). The main fluorescent coenzymes of bacterial respiration cycle are the reduced form of nicotinamide adenine dinucleotide (NADH) as well as flavins such as the oxidized form of flavin adenine dinucleotide (FAD) or its reduced forms. Their fluorescence lifetime and quantum efficiency (NADH: *τ*_1_ ~ 0.3 ns and QE ~ 2%; FAD: *τ*_1_ ~ 2 ns and QE ~ 3%) are weaker than those of the aromatic amino acids (Phenylalanine: QE ~ 4%; Tyrosine: QE ~ 21%; Tryptophane: QE ~ 20%) (Gorbunova et al., 2022).

NADH and FAD, as main co-enzymes in many metabolic enzymes, are non-invasive *in vivo* fluorescing probes of the metabolic status of the cell in biological systems in general (Chance et al., 1962). As an example in bacteriology, FAD auto-fluorescence imaging (Exc. 457 Em. > 495LP nm) has been used to study the effect of the CM15 antimicrobial peptide in a single *E. coli* cell (Choi et al., 2015). The authors observed that the spatial distribution was that of a filled cytoplasm and concluded that cellular auto-fluorescence was predominantly due to soluble flavins and flavin cofactors bound to soluble enzymes, not membrane-bound species. Yang et al. had already quantified green auto-fluorescence (Exc. 488 nm Em. 520BP35 nm) of *E. coli* cell in a laboratory-built high-sensitivity flow cytometer and attributed this auto-fluorescence to the oxidized FAD form of flavins (Yang et al., 2012). Several authors have studied the impact of different antibiotics on the FAD auto-fluorescence of bacteria in label-free experiments: Renggli et al. showed an effect of a 3 h incubation of Norfloxacin on *E.coli* (Renggli et al., 2013), Saint Ruf et al. demonstrated such an influence on auto-fluorescence of 3 antibiotics on *E.coli* (Saint-Ruf et al., 2016), and finally Surre et al. measured a similar effect on *E.coli* while showing that *de novo* protein synthesis was required for the observed auto-fluorescence increase (Surre et al., 2018). As a conclusion, the aforementioned label-free-cytometry-based trials confirmed the possibility to predict R/S phenotype based on single-cell scattering and auto-fluorescence, but focused only on Gram-negative bacteria, in particular *E. coli*, and investigated only partially the respiration-related auto-fluorescence, as only the FAD (i.e., green) channel was monitored while the NADH (i.e., blue) channel was not. Moreover, they were end-point-measurement-based trials and did not allow to evidence the potential transient nature of significant metabolic species.

In addition to fluorescence measurements, other works over the last decade suggested a strong correlation between the antibiotic effect and the bacterial respiration: not only the antibiotic perturbs the metabolic state of bacteria, but also the metabolic state of bacteria affects the antibiotic efficacy and lethality (Lobritz et al., 2015). For instance, an effect of antibiotic incubation has been measured on oxygen consumption of bacteria as well as on intracellular NADH concentration (Kohanski et al., 2007; Lobritz et al., 2015). The authors observed a transient depletion of NADH in *E. coli* after ~30 min of incubation with various bactericidal molecules, while no such impact was observed when using bacteriostatic molecules. The sum of the aforementioned auto-fluorescence, oxygen consumption and co-enzyme titration experiments indicate that NADH and FAD are promising endogenous probes to assess the susceptibility and resistance of bacteria to antibiotic molecules. Evaluating bacterial NADH and FAD through auto-fluorescence measurements for AST purpose is expected to have no impact on bacteria metabolism and to enable real-time monitoring while simplifying sample preparation.

Herein, we used the combination of the multi-parametric and high throughput advantages of flow cytometry probing light scattering and auto-fluorescence independently or in combination to evaluate the Susceptible/Resistant (S/R) phenotype of bacteria when exposed to antibiotic molecules. The originality of this work compared to previous label-free AST trials relies on:

- A biological model made of both Gram-negative (2 strains of *E. coli*) and Gram-positive (4 strains of *S. epidermidis*), as well as 3 bactericidal antibiotics, including beta-lactams and an aminoglycoside;
- An “all-in-culture-broth,” label-free and fixation-free protocol that enables the monitoring of bacteria in the presence/absence of antibiotics with a flow cytometer, over 2 h-long periods;
- The simultaneous access to four label-free parameters: two auto-fluorescence channels probing FAD and NADH, the main two co-enzymes involved in the bacteria cell respiration cycle, and the two classical forward and side scattering channels probing morphological and structural information, FSC and SSC;
- Two data analysis approaches involving dimensionality reduction of time-lapse cytometry data, aiming at comparing susceptible and resistant strain evolution, either at a population or at single-cell level.

This approach enabled us to show the feasibility of fast AST while relying exclusively on the intrinsic cellular characteristics of the micro-organisms.

## 2. Materials and methods

### 2.1. Bacterial strains

For our demonstration, we selected a Gram-negative model with *E. coli* (EC) and a Gram-positive model with *S. epidermidis* (SE). For each species, we selected both susceptible and resistant strains (6 in total) regarding a given list of antibiotics (see Table 1).

**Table 1:** Experimental design: list of antibiotic molecules and tested concentrations (including clinical breakpoints c and C) (CLSI, 2017), list of strains with their mode of resistance towards the antibiotic molecule, and measured minimal inhibitory concentration (MIC).

	Antibiotic	Antibiotic concentrations {0, c, C} (μg/mL)	Strain	Mechanism of resistance	MIC (μg/mL)
*Escherichia coli*	Amoxicillin	{0, 8, 32}	EC1 (ATCC25922)	Susceptible (Wild type)	8
			EC2 (ATCC35421)	Acquired penicillinase (hydrolysis of the antibiotic), (*SHV1*)	>64
	Gentamicin	{0, 4, 16}	EC1	Susceptible (Wild type)	0.5
			EC2	Enzymatic (antibiotic modification), (*ANT(2″), AAC(3)-II, AAC(3)-IV*)	>64
*Staphylococcus epidermidis*	Gentamicin	{0, 4, 16}	SE1 (API1501111, bioMérieux)	Susceptible (Wild type)	<0.31
			SE2 (API1501116, bioMérieux)	Susceptible (Wild type)	<0.31
			SE3 (API1501117, bioMérieux)	Enzymatic (antibiotic modification), (*APH(2″)* + *AAC(6′)*)	16
			SE4 (API1501118, bioMérieux)	Enzymatic (antibiotic modification), (*APH(2″)* + *AAC(6′)*)	>16
	Oxacillin	{0, 0.25, 0.5}	SE1	Susceptible (Wild type)	0.25
			SE2	Susceptible (Wild type)	0.125
			SE3	Modification of PBP (target modification), (*mecA*)	>16
			SE4	Modification of PBP (target modification), (*mecA*)	>16

### 2.2. Antibiotics and minimal inhibitory concentration

Three bactericidal antibiotics were selected: two beta-lactam molecules of the penicillin class that exhibit specific antibiotic effects against either EC (i.e., amoxicillin) or SE (i.e., oxacillin), and one aminoglycoside (i.e., gentamicin) that is efficient against both species. Gentamicin (Unipex solutions France) and oxacillin (TCI) were diluted with suspension medium (bioMérieux), and amoxicillin salt (Molcan Corporation) was diluted with Phosphate Buffered Saline 1X pH 7.4 (tablets Panreac AppliChem, diluted in milli-Q water). Antibiotics stock solutions were prepared once, according to the Clinical & Laboratory Standards Institute (CLSI) standard (CLSI, 2015); aliquots were stored at −20°C and used within a maximum of 6 months. Medium broth, buffers and antibiotics solutions were filtered on 0.2 μm sterile polyethersulfone membranes (VWR) to guaranty both sterility and absence of interfering particles and crystals. Minimal Inhibitory Concentrations (MIC) were measured via the broth micro-dilution method, according to the CLSI standard (CLSI, 2015) as well as in our specific experimental conditions, as described in the Supplementary Table S1. Breakpoints and MIC are listed in Table 1.

### 2.3. Flow cytometry measurements

Flow cytometry measurements were done directly in the incubation medium (i.e., MHB), in a special-order flow cytometer (LSRII, Becton Dickinson) equipped with a near-UV laser, at the SFR Biosciences (INSERM, Lyon, BSL1). We measured simultaneously the FSC and SSC scattering parameters (488 nm laser, measurement at 488/10 nm), as well as the green auto-fluorescence (Exc: 488 nm; Em: 525/50 nm) and blue auto-fluorescence (Exc: 355 nm; Em: 450/50 nm) parameters, whose excitation and emission range coincide with absorption/emission maxima of the FAD (Abs: ~450 nm; Em: ~535 nm), and NADH (Abs: ~340 nm; Em: ~460 nm) auto-fluorescence (Yang et al., 2012; Kolenc and Quinn, 2019), respectively. In the rest of the document the green and blue auto-fluorescence channels will be directly referred to as FAD and NADH. Cytometer settings are in the Supplementary Table S2. Acquisition stopped after 50′000 events. For each event and parameter, both area and height of the detected pulse were recorded but only height was used for further analysis.

### 2.4. Sample preparation and incubation

**Figure 1:**
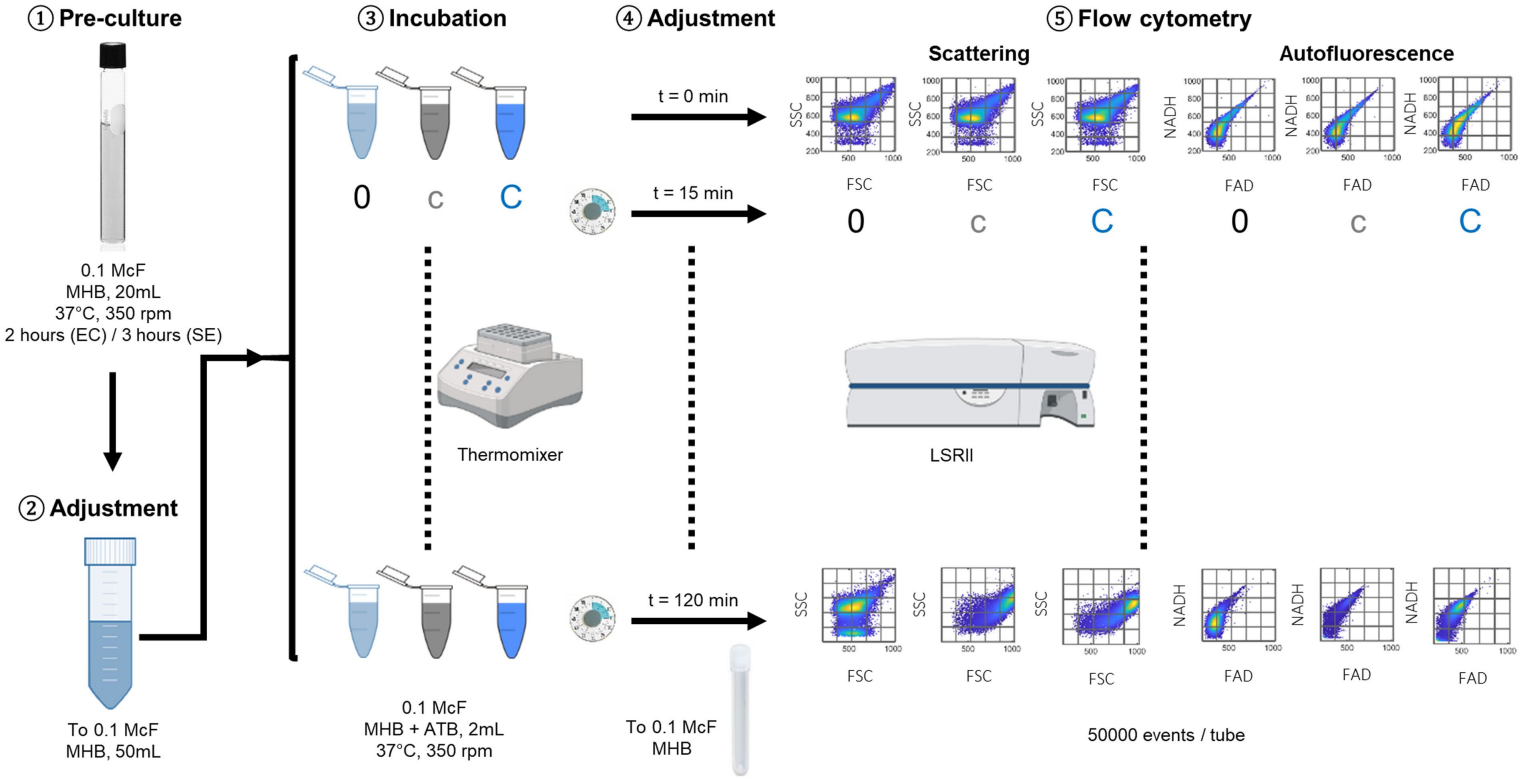
Daily experimental protocol, following an overnight culture on COS agar plate. ① Pre-culture in MHB, ② adjustment to 0.1 McF with fresh MHB, ③ start of incubation in 27 Eppendorf tubes at 37°C: 9 tubes without antibiotics (0), 9 tubes with the antibiotic molecule at the low breakpoint concentration (c), 9 tubes with the antibiotic molecule at the high breakpoint concentration (C); every 15 min, ④ readjustment to 0.1 McF with fresh MHB of one tube at 0, one tube at c, and one tube at C prior to ⑤ measurement in the flow cytometer: recording of 50′000 events per tube, in the FSC, SSC, FAD, and NADH channels.

Sample preparation is described in Figure 1. Bacteria were grown overnight at 37°C onto Columbia agar +5% sheep blood (COS, bioMérieux). In the morning, a 2 h (for *E. coli*) or 3 h (for *S. epidermidis*) pre-culture was cultivated in Mueller Hinton Broth (MHB, bioMérieux), at 37°C and under 250 rpm stirring. At *t*_0_, we diluted the pre-culture with fresh MHB to 0.1 McF ~ 3.10^7^ CFU/mL, and filled 27 sterile microcentrifuge tubes (Eppendorf, 2 mL). Tubes were then incubated either in absence of antibiotic (i.e., 9 tubes “0”), or in presence of the antibiotic at the low breakpoint concentration (i.e., 9 tubes “c”), or with the antibiotic at the high breakpoint concentration (i.e., 9 tubes “C”). Incubation took place in an incubator-shaker at 37°C and 350 rpm (Eppendorf, Thermomixer C, with ThermoTop) for maximum 2 h.

For each experiment, one tube “0,” one tube “c” and one tube “C” were analyzed using flow cytometry every 15 min over 2 h of incubation. At each time point, the three tubes were treated as follows. (1) The turbity was measured (Densimat, bioMérieux); when an increase of turbidity was observed compared to *t*_0_, the tube was diluted with fresh MHB back to 0.1 McF in order to standardize the subsequent flow cytometry measurements. (2) Twenty seconds vigorous vortexing of the tube was allowed in order to break possible aggregates and promote single bacterial cell analysis during the subsequent cytometry measurements. (3) Eventually, the flow cytometer recorded 50′000 events per tube and four parameters per event: two scattering parameters in the FSC and SSC channels, and two intrinsic fluorescence parameters in the FAD and NADH channels.

Both incubation and flow cytometry measurements were done directly in Mueller Hinton broth. This approach was selected to: (i) optimize the bacterial response to the antibiotic compared to an incubation in a minimum medium, (ii) use a medium used in most reference methods (e.g., in the broth micro-dilution method), and (iii) keep the sample-handling as simple as possible by avoiding washing steps. These choices enabled keeping a 15 min time-resolution for the time-lapse cytometry measurements all along the incubation, and limiting stresses to those induced by the antibiotic.

### 2.5. Control experiments

*Ctrl1*. The susceptible SE2 and the resistant SE3 strains were monitored w/o gentamicin while using an imaging flow cytometer ImageStream X (MarkII, Amnis, SFR Biosciences) instead of the LSRII. The time points 0, 60, and 120 min of incubation were analyzed.

*Ctrl2*. Auto-fluorescence of non-stained polystyrene beads (Sigma-Aldrich, ref. LB11, *ɸ* = 1.1 μm) were measured in water and in MHB, with the LSRII. The beads were also compared to the SE4 strain.

*Ctrl3*. The susceptible SE2 strain incubated w/o gentamicin was monitored with the LSRII after having washed the bacteria in a fluorescence-free buffer. More precisely, after incubation in MHB and turbidity measurement, the sample was centrifuged during 2.5 min at 2000 rcf in a microcentrifuge (Eppendorf 5,418). The pellet was then re-suspended in Dulbecco’s phosphate buffer saline (DPBS) to 0.1 McF prior to cytometry measurement.

### 2.6. Analysis with the subpopulation approach

A subpopulation approach was proposed to compare the data obtained from the different bacterial strains, over time and for different antibiotic concentrations, in order to assess whether the strains exhibit a behavior that can be attributed to their resistance phenotype. For this purpose we relied on Principal Component Analysis (PCA) (Hotelling, 1933) built from feature vectors summarizing the distributions of cytometry events obtained for the different concentrations and at various time points. More precisely, we constructed a 2D-binning in the FSC-SSC and FAD-NADH data by discretizing each of these 2D-spaces considering a fixed regular grid of size *n* × *n*, *n* being the number of bins per dimension. Each sample is then represented by two 2-D histograms comprising the proportions of events that fall in each bin when considering 50′000 events per sample. This is a 2D, static version of the more elaborated adaptive 3D-binning proposed by Huang et al. (2015). In our 2D-spaces, each dimension (i.e., each measured parameter) ranges between 200 and 1,025; *n* = 20 but a looser grid (i.e., *n* = 5) is shown on the figures for the sake of readability. In what follows, the 400-element vectors representing the FSC-SSC and FAD-NADH spaces were analyzed separately or after their concatenation in a 800-element vectors. PCA analysis was then performed independently for each strain and each of the three spaces (FSC-SSC, FAD-NADH and their concatenation), by summarizing the 27 experiments (i.e., 3 concentrations × 9 time-points) into matrices of size 27 × 400 or 27 × 800. In practice, the PCA analysis was implemented in R using the *prcomp* function from the stats package.

**Figure 2:**
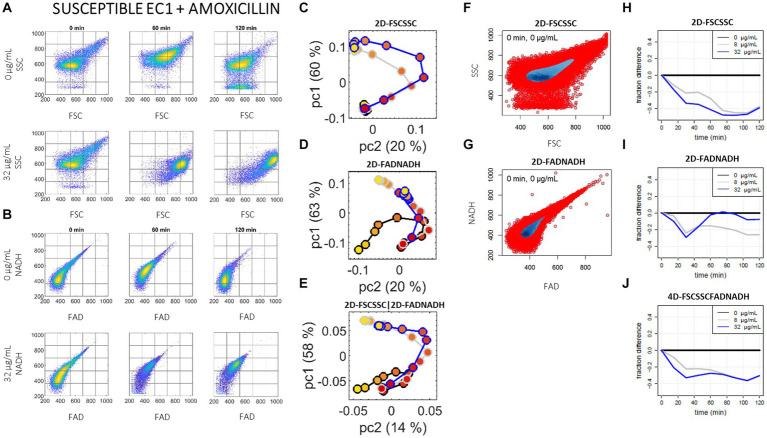
Illustration of the two data analysis pipelines, using a susceptible *E. coli* (EC1) incubated with amoxicillin. **(A,B)** 2D raw scatter plots. **(A–E)** Measurements are shown at 0 min (l-h-s), 60 min (center), and 120 min (r-h-s) of incubation, for 0 (first row) and the high breakpoint C = 32 μg/mL (second row) of amoxicillin: **(A)** raw SSC versus FSC scattering data, and **(B)** raw NADH versus FAD auto-fluorescence data. Each subplot shows 50′000 events, and the grid illustrates how 2D-distribution of events is eventually represented as a vector for the subsequent population-based PCA analysis (a 5 × 5 grid is shown here for clearer readability, but a 20 × 20 grid was used for the actual data analysis). **(C–E)** Population-based PCA analysis. Pc2 versus pc1 scores resulting from the PCA analysis of the 27 distribution vectors – nine time points (from red dots at 0 min to yellow dots at 120 min) and three amoxicillin concentrations, 0 μg/mL (black curve), c = 8 μg/mL (grey curve), C = 32 μg/mL (blue curve): **(C)** for the SSC versus FSC input scattering data, **(D)** for the NADH versus FAD auto-fluorescence input data, and **(E)** for the concatenated scattering and auto-fluorescence input data. **(F–J)**: Single-cell OC-SVM analysis. **(F–G)** Example of reference support (blue zone) determined by SVM analysis and containing 50% of the events at *t* = 0 min: **(F)** for the 2D scattering data at 0 μg/mL, and **(G)** for the 2D auto-fluorescence data at 0 μg/mL; a reference support is calculated at *t* = 0 min for each of the three antibiotic concentrations. **(H–J)** Fraction of events remaining in the reference support over time: **(H)** for the 2D scattering input data, **(I)** for the 2D auto-fluorescence input data, and **(J)** for the 4D scattering-auto-fluorescence input data (corresponding 4D reference supports not shown); at each time point, the fraction of in-support events for 0 μg/mL has been systematically subtracted.

Figure 2 illustrates the analysis workflow, taking as supporting example the susceptible *E. coli* (EC1) incubated without and with amoxicillin. Figures 2A,B shows the raw events distributions measured by flow cytometry. For the sake of readability, only two concentrations of amoxicillin (0 and C = 32 μg/mL) and three time points (0, 60, and 120 min) are shown. Figures 2C–E shows the scores of the first principal component (pc1) versus scores of the second principal component (pc2), for the FSC-SSC data (2C), for the FAD-NADH data (2D) and for their combination (2E). On these plots, each vector (representing 50′000 events) is represented by a single dot whose color stands for the time; lines link the dots to help following the evolution for a given concentration. During this study, the first two principal components could explain between 80% and 99% of the total variance of an experiment, depending on the studied incubation.

### 2.7. Analysis with the single-cell approach

A single-cell strategy was employed in order to investigate whether S/I/R phenotypes can be concluded from a random subset of events, with the objective to project the technology for direct sample (i.e., growth-free) diagnostics. For this purpose, a reference support of the event distributions in various spaces was estimated at *t*_0_ and for each antibiotic concentration. This reference support corresponds to a subset of the input space such that the probability that a test point lies outside of this support equals some *a priori* specified value between 0 and 1. We then monitor along time the fraction of events that fall within this reference support. If the antibiotic at the considered concentration does not have any effect on the monitored parameters, the cell distribution is expected to remain the same and therefore the proportion of events falling within the support to be constant over time. Conversely, if the antibiotic at the considered concentration has an effect on the parameters, the shift of the event distribution will translate into a lower number of events within the reference support. By considering such fraction curves for different antibiotic concentrations, we can quantify the effect of the antibiotic and conclude about the strain resistant or susceptible phenotype.

To estimate the reference support, we rely on One-Class Support Vector Machine (OC-SVM) (Schölkopf et al., 2001). Given a set of samples, the OC-SVM algorithm allows estimating the support of the underlying distribution for these samples in a discriminative manner: it takes as input a parameter *nu* between 0.1 and 1 and a “kernel” function, which defines the nature of the support boundary (e.g., whether it is linear or not), and seeks for a support containing the (1-*nu*) fraction of points of the reference distribution. The *nu* parameter therefore controls the fraction of points that we accept to leave outside of the support: the smaller *nu*, the larger the support. Herein *nu* = 0.5 to focus on half of the initial events, and a radial basis kernel was used. In practice, we used the *e1071*^1^ R package to implement the OC-SVM single-cell analysis. Figures 2F–J illustrate the single-cell analysis workflow. An example of the reference supports in the case of no antibiotic are shown for the 2D FSC-SSC space (2F) and the 2D FAD-NADH space (2G); the 4D FFS-SSC-FAD-NADH space is not shown. Figures 2H–J show the resulting fraction curves at the three antibiotics concentrations as a function of time, for the FSC-SSC space (2H), the FAD-NADH space (2I), and the 4D FSC-SSC-FAD-NADH space (2J). For the sake of readability, the fraction curve at 0 μg/mL was subtracted; as a result, the “no antibiotic” final curve (in black) is systematically at *y* = 0 and the “c” (in grey) and “C” (in blue) curves represent both the evolution compared to t_0_ but also to the no antibiotic condition.

## 3. Results

Determining the resistance phenotype of a given strain regarding a given antibiotic consists in categorizing the germ as either susceptible (S), intermediate (I) or resistant (R) during *in vitro* incubation with that antibiotic. S/I/R categorization is ruled by the low (c) and high (C) breakpoint concentrations so that MIC ≤ c for S strains, c < MIC < C for I strains, and C ≤ MIC for R strains (CLSI, 2017). When compared to the breakpoints, the measured MIC show that the EC1, SE1, and SE2 strains are susceptible to the selected molecules, and that EC2, SE3, and SE4 are resistant (Table 1). Interestingly, the current experimental conditions do not have a significant impact on MIC measurements compared to CLSI standard (Supplementary Table S3). Three antibiotic concentrations were simultaneously tested for each combination strain/antibiotic: 0, c, and C. In total 12 combinations of strain/antibiotic were studied; duplicates always took place at different dates (Table 1). In order to analyze the large amount of data that are generated in one experiment (i.e., 3 antibiotic concentrations, 9 time points, 50′000 events per concentration and time-point, 4 parameters per event), two approaches of dimensionality reduction (at sub-population, or at single cell levels) were tested: a sub-population approach and a single-cell approach. Each of them offers a different graphical representation aiming at discriminating between susceptible and resistant strains as a function of time. Using both analysis approaches, we assessed scattering and auto-fluorescence parameters separately and in combination with each other.

### 3.1. *Escherichia coli* and beta-lactam

**Figure 3:**
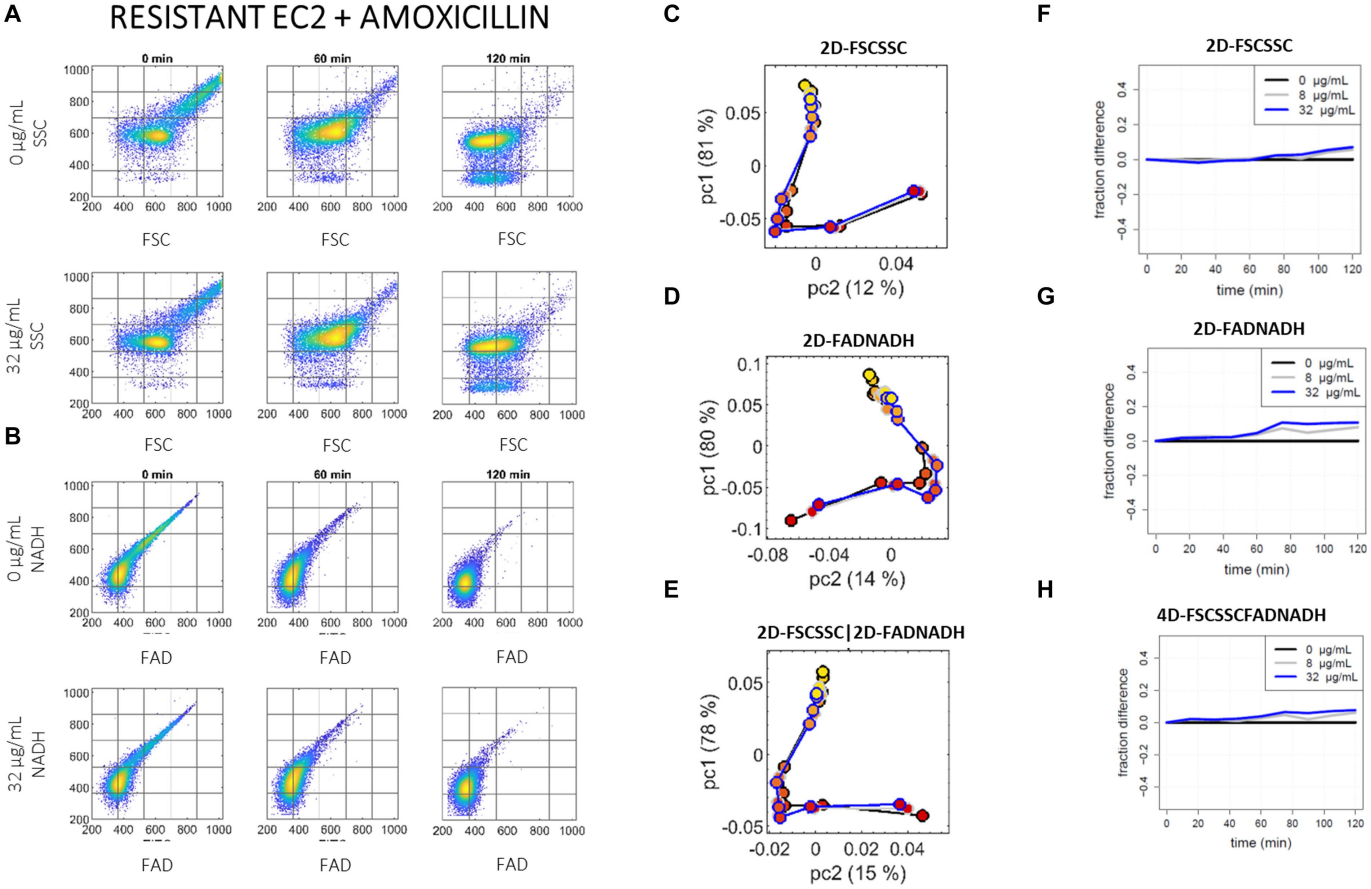
Illustration of the data set and analysis results for the resistant *E. coli* (EC2) incubated with amoxicillin. **(A,B)**: 2D raw scatter plots. Measurements are shown at 0 min (l-h-s), 60 min (center), and 120 min (r-h-s) of incubation, for 0 (first row) and the high breakpoint C = 32 μg/mL (second row) of amoxicillin: **(A)** raw SSC versus FSC scattering data, and **(B)** raw NADH versus FAD auto-fluorescence data. **(C–E)** Population-based PCA analysis. Pc2 versus pc1 scores resulting from the PCA analysis of the 27 distribution vectors – nine time points (from red dots at 0 min to yellow dots at 120 min) and three amoxicillin concentrations, 0 μg/mL (black curve), c = 8 μg/mL (grey curve), C = 32 μg/mL (blue curve): **(C)** for the SSC versus FSC input data, **(D)** for the NADH versus FAD input data, and **(E)** for the concatenated scattering and auto-fluorescence input data. **(F–H)**: Single-cell OC-SVM analysis. Fraction of events remaining in the reference support over time: **(F)** for the input 2D scattering data, **(G)** for the input 2D auto-fluorescence data, and **(H)** for the input 4D scattering-auto-fluorescence data; at each time point, the fraction of in-support events for 0 μg/mL has been systematically subtracted.

Figure 2 shows the case of the susceptible EC1 strain when incubated without (0 μg/mL) and with amoxicillin (at the high breakpoint C = 32 μg/mL), to be compared with the case of the resistant EC2 strain shown in Figure 3. When comparing the raw event distributions (A) and (B) of Figures 2, 3, untreated EC1 and EC2 showed similar scattering and auto-fluorescence distribution patterns. However, a significant impact was observed on the susceptible strain upon amoxicillin treatment. Indeed, a shift of the denser event region towards higher scattering and higher fluorescence parameters was observed while no impact was observed on the resistant strain distribution over the 2 h incubation. The shift in scattering is in agreement with the well-documented elongation and “bulging” of EC upon exposure to a molecule of the penicillin class (Comber et al., 1977; Gottfredsson et al., 1998; Yao et al., 2012; Nonejuie et al., 2013; Cushnie et al., 2016). Scatter plots for the untreated strains naturally evolve over time too, but at a much smaller extent; this evolution was not monotonic and stabilized before the end of all experiments, for both the susceptible and resistant EC. This behavior may relate to the time the bacteria need to adapt to the contact with fresh medium at *t*_0_ and reflect the natural evolution of a dividing strain.

The analyzed data for the susceptible and resistant EC strains, first at the sub-population approach, can be compared via the pc1 versus pc2 plots (C)–(E) of Figures 2, 3. The susceptible EC1 exhibits a clear divergence of trajectories over time (i.e., from red to yellow dots) between the curves with and without amoxicillin either from the 2D scattering data, the 2D auto-fluorescence data or their combination; on the other hand, the resistant EC2 trajectories are superimposed in the presence or absence of antibiotic in all three spaces. A clear S/R phenotyping is thus possible from the label-free cytometry data from the first 2–3 time points but no specific benefit of the auto-fluorescence parameters is noticed in this case, just the scattering parameters allow for a clear phenotype prediction.

Results of the single-cell approach for the susceptible and resistant EC strains can be compared via the fraction curves shown on Figures 2H–J, 3F–H, respectively. For the susceptible EC1 strain under amoxicillin treatment, a rapid decrease of the fraction curves occurs in all three spaces (compared to the no antibiotic condition), which is consistent with the immediate shift of the raw event distributions outside of the reference support estimated by the OC-SVM. While the divergence increases regularly on FSC-SSC (up to *y* ~ −0.5 at 1 h), the divergence observed at first on FAD-NADH (up to *y* ~ −0.3 at 30 min) diminishes after 30 min. This transitory behavior during the first hour of incubation reminds on the transitory relative [NADH] drop measured by Kohanski et al. in wild type *E. coli* following treatment with norfloxacin, ampicillin or kanamycin (Kohanski et al., 2007). On the other hand, the resistant EC2 shows no divergence between the control and the antibiotic curves for the first 45 min; a slow divergence appears after 60 min on FSC-SSC (*y* ~ 0.1 at 2 h) and after 45 min on FAD-NADH (plateau at *y* ~ 0.1 after 90 min). Through this second analysis approach we confirm that for EC and amoxicillin a clear S/R phenotyping is quasi instantaneously possible from the label-free cytometry data, and that no benefit of auto-fluorescence measurement is observed for that purpose. Comparison of the current OC-SVM results with that of duplicate experiments at different days are available in the Supplementary Figures S1A,C: while absolute values are not strictly comparable from day to day, the global difference observed between the susceptible EC1 and the penicillinase-producer EC2 remains as striking on the duplicate.

### 3.2. *Escherichia coli* and aminoglycoside

For the interested readers, an example of the raw event distributions measured during one experiment of the susceptible EC1 and one experiment of the resistant EC2 without and with gentamicin (at the high breakpoint) are shown in the Supplementary Figure S2. One can note that the distributions of the susceptible EC1 treated with gentamicin (Supplementary Figures S2A,B) shows a very different evolution with time compared to the previous case (amoxicillin, Figures 2A,B): while amoxicillin induced a shift of FSC-SSC and FAD-NADH scatter plots very significantly and rapidly, gentamicin had a freezing impact on the distributions of both scatter plots. The observed freezing effect of gentamicin on the scattering distribution of EC1 is in agreement with the impact of aminoglycosides on the morphology of *Escherichia coli*, as reported in several microscopy analyses (Nonejuie et al., 2013; Bruni and Kralj, 2020; Aguirre Rivera et al., 2021).

**Figure 4:**
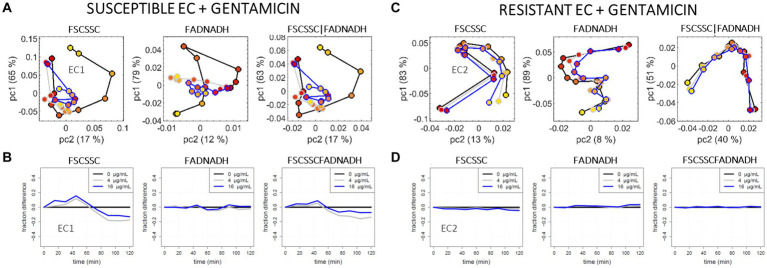
PCA and OC-SVM analysis of the cytometry data recorded for the *E. coli* + gentamicin model: the susceptible EC1 strain **(A,B)** versus the resistant EC2 strain **(C,D)**, for gentamicin at 0 μg/mL (black curves), c = 4 μg/mL (grey curves) and C = 16 μg/mL (blue curves). **(A,C)** pc2 versus pc1 scores, for the 2D scattering data (l-h-s), the 2D auto-fluorescence data (center), and the concatenation of the 2D scattering and the 2D auto-fluorescence data (r-h-s). **(B,D)** Fraction of events remaining in the reference (*t* = 0 min) SVM support over time, for the 2D scattering data (l-h-s), the 2D auto-fluorescence data (center), and the 4D scattering-auto-fluorescence data (r-h-s).

Figure 4 compares the analyzed data for the susceptible EC1 and the resistant EC2. Results of the sub-population approach are shown in Figures 4A,C. Different trajectories are visible between the treated (grey and blue curves) and untreated (black curve) cases for EC1 (4A), while the resistant EC2 exhibits quasi superimposed trajectories for all cases (4C). It is interesting to see that in the present case (i.e., gentamicin), the untreated EC1 trajectories evolve more than the treated ones, which is the opposite of the observation made for the previous case (i.e., amoxicillin). However, S/R phenotyping remains possible when comparing EC1 and EC2 trajectories, and scattering data seem to suffice for that purpose.

On the representation of the single-cell approach (Figures 4B,D), divergence between the treated and untreated EC1 curves (4B) is clearly visible only for the scattering data; when comparing EC1 (4B) and the resistant EC2 curves (4D) one can easily deduce the S/R phenotype based on scattering. Comparison of the current results with that of duplicate experiments at different days are available in the Supplementary Figures S1B,D. In spite of absolute discrepancies between the replicated incubations of the susceptible strain, the global difference observed between the susceptible EC1 and the resistant EC2 remains true for the duplicate.

### 3.3. *Staphylococcus epidermidis* and aminoglycoside

Examples of the raw event distributions for the case SE (susceptible SE2 and resistant SE3) incubated without and with gentamicin are available in the Supplementary Figure S3. The untreated SE strains naturally evolve over time, as observed previously for the untreated EC strains, but this time in a monotonous way over the 2 h, with a denser zone of events shifting towards smaller values for the 4 measured parameters (FSC, SSC, FAD, and NADH). When a treatment with gentamicin is applied, the resistant strain evolves as if no treatment was applied, while the susceptible strain evolves much less in comparison, as previously observed for the susceptible EC treated with gentamicin. The freezing impact of gentamicin on the scattering distribution of the susceptible SE strain is due to the impaired protein replication, which stops the division and part of the metabolism. It can be compared to the absence of structural changes observed by Scanning Electron Microscopy (SEM) on susceptible *S. aureus* treated with gentamicin (Rýc and Výmola, 1986). The impact of gentamicin on the FAD-NADH event distribution is much less visible than it is in the FSC-SSC space.

**Figure 5:**
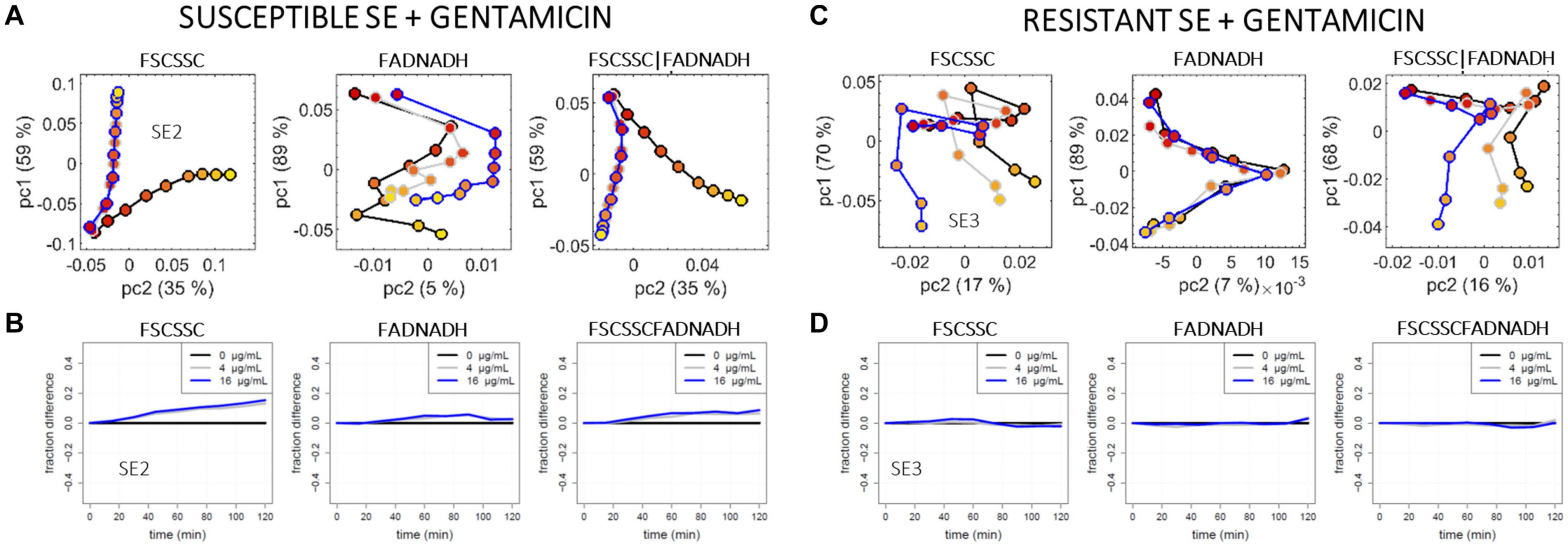
PCA and OC-SVM analysis of the cytometry data recorded for the *S. epidermidis* + gentamicin model: the susceptible SE2 strain **(A,B)** versus the resistant SE3 strain **(C,D)**, for gentamicin at 0 μg/mL (black curves), c = 4 μg/mL (grey curves) and C = 16 μg/mL (blue curves). **(A,C)** pc2 versus pc1 scores, for the 2D scattering data (l-h-s), the 2D auto-fluorescence data (center), and the concatenation of the 2D scattering and the 2D auto-fluorescence data (r-h-s). **(B,D)** Fraction of events remaining in the reference (*t* = 0 min) SVM support over time, for the 2D scattering data (l-h-s), the 2D auto-fluorescence data (center), and the 4D scattering-auto-fluorescence data (r-h-s).

Figure 5 compares the analyzed data for the susceptible SE2 and the resistant SE3. Results of the sub-population approach are shown Figures 5A,C. Contrarily to the previous EC case, one does not see a strict superimposition of treated and untreated trajectories of the resistant SE strain. However, S/R phenotyping remains possible as the “0” and “C” trajectories diverge in a different way for SE2 (5A) and SE3 (5C); this difference is particularly noticeable in the FSC-SSC space.

On the single-cell approach representation (Figures 5B,D), divergence between the treated and untreated SE2 curves (5B) is visible in both scattering space (*y* ~ 0.2 at 2 h) and auto-fluorescence space (0 < *y* < 0.1), while a negligible divergence (|*y*| < 0.05 over 2 h) is seen for the resistant SE3 strain (5D). The reason for the antibiotic impact on the susceptible strain being less pronounced in the auto-fluorescence space than in the scattering space could be the parasitic auto-fluorescence of the surrounding MHB. One notes the smoother evolution of the SE curves compared to that of the EC curves (shown in Figure 4); this may be related to the spherical symmetry of cocci which makes the measurement of the height of each pulse/event more robust compared to the rod-shape symmetry of bacilli. A comparison of the current OC-SVM results with that of duplicate experiments are available in the Supplementary Figures S4A,C. In order to reinforce the demonstration, two other strains (i.e., the susceptible SE1 and the resistant SE4) were studied and compared to the first 2 strains (see Supplementary Figures S4B,D for OC-SVM results and Supplementary Figure S5 for the raw event distributions), leading to the same general conclusion: for the case SE and gentamicin, S/R phenotyping is easier from scattering data than from auto-fluorescence data.

Of note, the control experiment *Ctrl1* for which an imager flow cytometer was used confirmed that, in SE + gentamicin incubations, 95% to 98% of the measured events were single cocci and diplococci while aggregates were a minority (see Supplementary Figure S6).

### 3.4. *Staphylococcus epidermidis* and beta-lactam

Examples of the raw event distributions for the case SE (susceptible SE1 and *mecA*+ SE3) without and with oxacillin are available in the Supplementary Figure S7. As seen previously, a natural evolution of the untreated strains occurs over time. While comparing the conditions 0 and C of the susceptible SE1, one sees that oxacillin affects noticeably the distribution of both the scattering and the auto-fluorescence parameters of the susceptible strain. However, an impact of oxacillin is also observed in the scattering space of the resistant strain SE3, contrarily to what has been seen above for the resistant EC. In comparison to scattering, the impact of oxacillin on the auto-fluorescence of SE3 is almost negligible. This behavior has been confirmed for two other strains in similar conditions, the susceptible SE2 and the *mecA*+ SE4 (Supplementary Figure S8). The impact of oxacillin on the scattering of both the susceptible and the *mecA*+ SE strains can be related to the morphological and structural impact of oxacillin, as it has been reported by SEM on both susceptible and *mecA*+ strains of *S. aureus* (Rýc and Výmola, 1986; Deshmukh et al., 2021).

**Figure 6:**
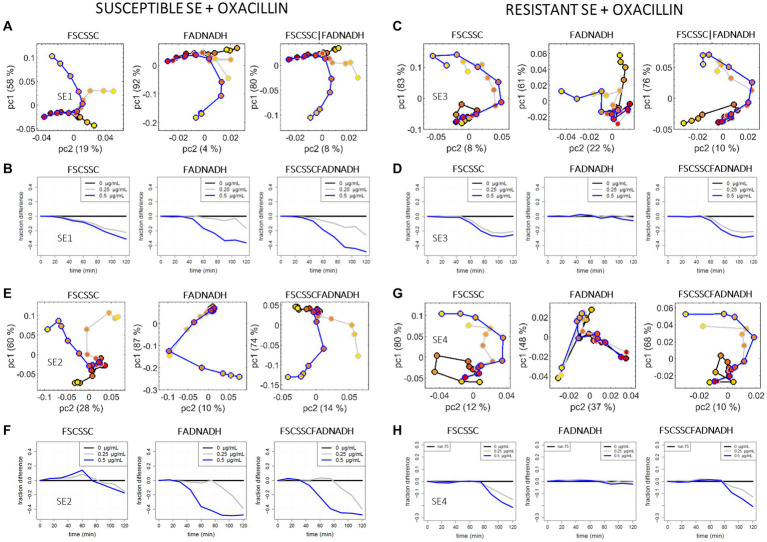
PCA and OC-SVM analysis of the cytometry data recorded for the *S. epidermidis* + oxacillin model: the susceptible SE1 strain **(A,B)**, the resistant SE3 strain **(C,D)**, the susceptible SE2 strain **(E,F)**, and the resistant SE4 strain **(G,H)**, for gentamicin at 0 μg/mL (black curves), c = 0.25 μg/mL (grey curves), and C = 0.5 μg/mL (blue curves). **(A,C,E,G)** pc2 versus pc1 scores, for the 2D scattering data (l-h-s), the 2D auto-fluorescence data (center), and the concatenation of the 2D scattering and the 2D auto-fluorescence data (r-h-s). **(B,D,F,H)** Fraction of events remaining in the reference (*t* = 0 min) SVM support over time, for the 2D scattering data (l-h-s), the 2D auto-fluorescence data (center), and the 4D scattering-auto-fluorescence data (r-h-s).

Figure 6 compares the analyzed data for the susceptible SE1 and the *mecA*+ SE3 when incubated with and without oxacillin. Results of the sub-population approach are shown Figures 6A,C. On this representation, a discrepancy is visible between the treated and untreated SE1 curves (6A) using both scattering and auto-fluorescence spaces data analyses. However, discrepancies are also observed with the *mecA*+ strain (6C). The behavior observed in the scattering space is in accordance with what has been observed directly on the raw data (Supplementary Figure S7C). However, the PCA representation in the auto-fluorescence space of the resistant strain also captures some divergence, which was not clearly perceptible when looking at the raw distribution (Supplementary Figure S7D). In this case, it is important to refer to the single cell OC-SVM representation, in particular to have a better view of the time scale of the significant divergences between the curves of the treated and untreated bacteria.

With the single-cell OC-SVM approach (Figures 6B,D), a divergence between the treated and untreated SE1 curves (6B) is observed in both scattering and fluorescence spaces after 30 min and in similar proportions (between −0.3 < *y* < −0.35 at 2 h). A divergence (*y* ~ −0.3 at 2 h) is also observed on the scattering data of the *mecA*+ strain (6D), confirming the observations made on the raw data and PCA analysis. However, the current representation reveals that the impact of the incubation appears a bit later on the resistant strain (45 min) than on the susceptible strain (30 min). A subtle time delay seems to be the only discriminating factor between the susceptible and the resistant strains if looking at the scattering parameters only. On the other hand, on this representation the divergence of the auto-fluorescence curves for the treated and untreated *mecA*+ strain is negligible over the course of the experiment (|*y*| < 0.05 over 2 h), marking a real difference with the behavior of the susceptible strain (*y* ~ −0.35 at 2 h). Our data suggest that, for the current case, auto-fluorescence allows a better differentiation between susceptible/resistant phenotypes than scattering alone. In the auto-fluorescence space, the phenotyping is based on a simple presence/absence of divergence between the treated and untreated curves, while in the scattering space the phenotyping may be extrapolated on the basis of a subtle time delay observed between susceptible and resistant bacterial isolates.

In order to reinforce the demonstration, two other SE strains have been studied: the susceptible SE2 and the *mecA*+ SE4. Raw event distributions are shown on Supplementary Figures S7, S8, while PCA and OC-SVM analyses are shown on Figures 6E–H. When focusing on the OC-SVM representation, the same general observation is true for the two susceptible strains on one side and the two resistant strains on the other side. First, oxacillin affects the scattering of both the susceptible and resistant SE strains in a similar way; the only subtle difference between the resistant and the susceptible strains is a time delay in the appearance of the effect of oxacillin. On the other hand, from the auto-fluorescence point of view, amoxicillin significantly affects only the susceptible SE strains over the 2 h incubation. In such case, it could be difficult to base S/R phenotyping on the scattering observations, while auto-fluorescence have a real benefit for the diagnostic.

## 4. Discussion

In this work we demonstrated the feasibility of fast (<2 h) antibiotic susceptibility testing based on scattering and auto-fluorescence parameters of single bacteria cells measured in a label-free flow cytometry mode. The originality of our work is based on four main factors.

### 4.1. Gram-negative and gram-positive bacteria model

Herein we showed that fast AST using label-free flow cytometry is possible for the entire biological models that were tested. The method developed in this study is promising as it shows good reproducibility and is consistent across multi-strains.

Antibiotics belonging to two inhibitor families were tested: inhibitors of cell wall synthesis with two beta-lactam molecules (amoxicillin and oxacillin), and one inhibitor of protein biosynthesis with the aminoglycoside molecule (gentamicin). Doing so we included some diversity in mechanisms of action of the tested antibiotics. Moreover, drug susceptibility assays included both Gram-negative and Gram-positive species to provide a more comprehensive demonstration. In particular, gathering data with *mecA*+ *S. epidermidis* revealed a new behavior and conclusions compared to previous studies based on Gram-negative species.

On the other hand, the tested EC strains (Gram-, bacillus) featured less smooth curves and more variability than SE (Gram+, coccus) strains, but antibiotic impact on susceptible strains was more striking on EC than on SE, especially when treated with beta-lactams. Interestingly, our experiments with SE and oxacillin showed very different behavior compared to other species/antibiotic combinations: oxacillin affected the susceptible and resistant (*mecA*+) strains in a very similar way, from the scattering point of view, while it affected the auto-fluorescence intensity of the two strains in a significantly different way. This case showed that scattering (commonly related to cell morphology and structure) is not always sufficient for fast AST and that auto-fluorescence is an interesting additional information to consider. The scattering change in the susceptible SE strains may be attributed to the same morphological and structural changes observed on susceptible *S. aureus* in the presence of oxacillin, as observed by SEM; in this case, cell division inhibition was showed to correlate with an increase in cross-wall and a decrease of peripheral cell wall thicknesses (Rýc and Výmola, 1986). The impact of oxacillin on the scattering parameters of the resistant SE strains may be linked to the *mecA* resistance gene. This gene codes for the penicillin-binding protein 2 (PBP-2) that has a low affinity for the beta-lactams, thus conferring the strain with a resistance to this class of molecules. However, this characteristic is also known to result in a modified growth pattern, also affecting the morphology and structure of the bacterial cells and – as a consequence – their scattering parameters. Such a structural change could be observed by SEM on *mecA*+ *S. aureus* (Deshmukh et al., 2021). On the other hand, the penicillinase of the resistant *E. coli* degrades the antibiotic molecule directly, which may explain why the morphology and structure of the resistant EC strain are not impacted (so as their single cell scattering parameters).

### 4.2. An “All In broth” simplified protocol in flow cytometry

We performed all the incubations in MHB in order to boost the bacterial response to the antibiotic compared to an incubation in a minimum medium, and to use a medium used in most reference methods. Not washing MHB prior to cytometry measurements was a deliberate choice aiming at keeping the sample handling as simple as possible, reducing the delay between each time points to 15 min and limiting bacterial stress.

In the current study, the auto-fluorescence of MHB more than likely affected the sensitivity on the auto-fluorescence parameters, as illustrated by the results of the control experiment *Ctrl2* (Supplementary Figure S9). Fluorescence of non-stained polystyrene beads showed that emissions in the FAD and NADH channels increase, respectively, by a factor of 3.5 and 14 when MHB is used instead of water (S9A). As a result, the SE4 bacterial cells (the only strain tested in those conditions) and the non-stained polystyrene beads (of similar size and morphology) show similar FAD signals in MHB; however different levels of signals are visible in the NADH channel (S9B). In spite of the MHB interference, we were able to measure informative bacterial auto-fluorescence signals in the current study. Washing the bacteria in PBS prior to flow cytometry during experiment *Ctrl3* had a positive impact on the auto-fluorescence sensitivity (Supplementary Figures S10, S11), as divergence between the “0” and “C” curves were at least twice larger in PBS than in MHB. However, the washing also had a negative impact on the smoothness of the curves as a function of time, for both scattering and auto-fluorescence measurements. As washing the MHB at each time point could increase stress and delay between the time points, and more generally add a source of variability, we did not retain this step for our current protocol. One could instead envisage limiting the environment auto-fluorescence by performing the incubation in diluted MHB to reduce those adverse effects but still enhance the signal-to-nose ratio.

### 4.3. Two analysis approaches of time-lapse cytometry

In the current study, we performed time-lapse cytometry measurements (i.e., every 15 min for 2 h) rather than end-point measurements. This enabled us to compare with previous time-lapse studies based on extraction procedures, which showed a potentially drastic evolution of NADH concentration in the first hour following exposition with bactericidal antibiotics (Kohanski et al., 2007). We proposed two ways of analyzing data in order to reduce dimensionality of data and capture the global evolutions of the scatter plots over time as a function of the antibiotic concentration. Doing so, we could compare divergence of trajectories without and with antibiotic, for the susceptible and resistant strains. The conclusion is that S/R phenotyping (i.e., AST) by label-free flow cytometry is possible in less than 2 h through dynamic monitoring of scattering and auto-fluorescence.

The sub-population approach “binning + PCA” enables detecting fine changes in scattering and fluorescence distribution trajectories. However, in the principal component space, we saw that trajectories are not necessarily smooth. Time-related variations can be cumbersome to interpret because time is not shown on a linear axis. Moreover, the comparison of axis is not possible from one graph to another: pc1 and pc2 scores are appropriate within a unique run, but quantitative scores cannot be compared across unrelated experiments; the characterization of the divergence of trajectories can only be qualitative. Nevertheless, the simplified sub-population approach was in most cases sufficient to appreciate a susceptible/resistance phenotype (Figures 3–5). It was more difficult for the SE + oxacillin study (Figure 6).

In contrast, the single-cell OC-SVM approach allows comparing the results across unrelated experiments because it relies only on relative fractions (fraction of events within the reference support) and a time-dependent representation on the *x*-axis. It exhibits smoother time profiles, and enables clear susceptible/resistant phenotyping in all tested cases. The presented 2D scatter plots are made of 50′000 events/bacteria cells, and the OC-SVM approach we presented herein still uses 50% of the initial events as a reference support (*nu* = 0.5). The resulting curve plots represent an ideal scenario in which we could assess a great number of cells. The next step of the analysis would be to draw random samples of 10’s or 100’s of cells and assess the minimum number of cells allowing the successful susceptibility testing. This would tell how such predictions could be used for the characterization of clinical samples with reduced bacterial loads.

### 4.4. Two auto-fluorescence channels on top of scattering

Auto-fluorescence was measured in the green (Exc: 488 nm; Em: 525/50 nm) and blue (Exc: 355 nm; Em: 450/50 nm) channels, associated to the FAD and NADH coenzyme signals, respectively. However, other molecules are susceptible to be simultaneously detected in the same channels. For instance, Nicotinamide Adenine Dinucleotide Phosphate (NADPH) is a potential emission competitor in the blue auto-fluorescence channel. NADPH is more specifically involved in the anabolic pathways of the bacterial metabolism while NADH is involved in catabolic reactions (Alberts et al., 2002). NADH and NADPH are spectrally similar to each other (both absorption maxima around 340 nm and emission maxima around 445–460 nm) and present comparably low fluorescence quantum yields. Therefore, they may be detected simultaneously in the blue auto-fluorescence channel. However, titration studies of the two metabolites in EC report bacterial intracellular NADH pool to be 3–10 times higher than the NADPH pool (Andersen and von Meyenburg, 1977), which probably limits the extent of that competition. Semiquinone (FADH^−^) and hydroquinone (FADH_2_) forms of Flavin Adenine Dinucleotide could also be potential emitters in the blue auto-fluorescence channel, if the UV excitation enables some partial excitation of these species (Zhao et al., 2011). Regarding the green auto-fluorescence channel, Yang et al. concluded from a dithionite reduction experiment that the signal they measured (Exc: 488; Em: 520/35 nm) originated from flavins auto-fluorescence (Yang et al., 2012). Surre et al. also used reporters to measure expression of ribA, ribB, ribC, and ribE, which are essential genes required for Flavin mononucleotide (FMN) and FAD biosynthesis, and observed that the gene expression increased significantly in cells treated with ampicillin, while the green auto-fluorescence was increasing (Exc: 488; Em: 525/20 nm) (Surre et al., 2018). It is thus reasonable to consider that flavins are major contributors to the observed auto-fluorescence increase in the green region in our experiments.

The auto-fluorescence increase in the green channel under antibiotic treatment may be due to “an increased expression of genes encoding diverse flavoproteins which are involved in energy production and Reactive Oxygen Species (ROS) detoxification, which indicates a cellular strategy to cope with severe stresses” (Surre et al., 2018). Kohanski et al. showed that the three major classes of bactericidal antibiotics with different targets stimulate the production of highly deleterious hydroxyl radicals in Gram-negative and Gram-positive bacteria, which ultimately contributes to cell death (Kohanski et al., 2007). They demonstrated that the mechanism of hydroxyl radical formation induced by bactericidal antibiotics is the end-product of an oxidative damage cellular death pathway involving the Tricarboxylic Acid Cycle (TCA), a transient depletion of NADH, a destabilization of iron–sulfur clusters, and a stimulation of the Fenton reaction. Following an exposure of *S. epidermidis* with beta-lactams (in particular oxacillin), Thomas et al. confirmed a contribution of the TCA-dependent ROS in enhancing susceptibility (Thomas et al., 2013). This study also suggests that the increased protection from beta-lactams (in resistant strains) could result from pleiotropic effects of a dysfunctional TCA cycle, including increased resistance to oxidative stress, reduced susceptibility to autolysis, and a more positively charged cell surface.

In the present work, we measured both scattering and auto-fluorescence parameters by flow cytometry. The pulse recorded at each event passing into the laser beam describes the integrated parameter over the entire bacterium, hence an incomplete decorrelation between the auto-fluorescence and the size of the bacterial cell (which is related to scattering). Surre et al. suggested to decorrelate the auto-fluorescence and morphology by dividing the measured integrated auto-fluorescence by the estimated volume of the bacteria cell (Surre et al., 2018). The case of *E. coli* plus beta-lactam has been studied before by label-free flow cytometry, showing a concomitant increase of scattering and green auto-fluorescence, which is also what we observed (Renggli et al., 2013; Surre et al., 2018). Note that using other antimicrobials, for instance with the antimicrobial peptide CM15, the increase of flavins auto-fluorescence can be correlated with a shrinkage of the *E. coli* cells (Choi et al., 2015). In the current study, the added-value of monitoring the auto-fluorescence on top of the scattering parameters is particularly clear in the case of SE + oxacillin. In this case, the increase of scattering parameters was not correlated with any significant increase in auto-fluorescence, suggesting that only the auto-fluorescence was sufficient to differentiate the S and R phenotypes.

Previous published studies only documented the green auto-fluorescence channel. In a last analysis shown in the Supplementary material, we assessed individually each of the four measured parameters for S/R phenotyping rather than considering them in 2D or 4D spaces (Supplementary Figures S12–S19). In particular, when comparing the contributions of the green auto-fluorescence (i.e., FAD) and of the blue auto-fluorescence (i.e., NADH) separately, NADH may be slightly more efficient than FAD for S/R phenotyping but it does not appear to be a mandatory information, at least for the biological model presented herein. Based on the current biological model, the use of a cytometer equipped with a near-UV laser does not seem to be mandatory.

## 5. Conclusion

We explored the use of scattering and auto-fluorescence parameters measured at the single cell level for fast, label-free S/R phenotyping. We proposed a flow cytometry-based tool, enabling high statistic and S/R phenotyping results in less than 2 h, i.e., in a much shorter time compared to most (low sensitivity, growth-detection-based) phenotypic diagnostic tests. The demonstration was made by monitoring scattering and auto-fluorescence parameters of 50′000 single bacteria cells per sample. We applied an original statistical analysis approach based on one-class Support Vector Machine, to interpret and represent the large amount of cytometry data in an easy-to handle way. Compared to growth-based methods such as classical optical density measurements, our single cell approach enables measurements in samples that contain a limited biomass.

Moreover, to our knowledge, this work is the first proof of concept of an antibiotic susceptibility test in less than 2 h using label-free flow cytometry considering both Gram-negative and Gram-positive species. The interest in monitoring auto-fluorescence parameters when working at the single cell level appeared when *mecA* mechanism of action was involved, leading to morphological and structural changes in *mecA*− and *mecA*+ bacteria isolates exposed to oxacillin. Considering the biological model in our study, FAD auto-fluorescence was informative enough to predict phenotype while NADH fluorescence was not mandatory but remained informative in itself.

The study focused on biosafety-level-1 strains. As a perspective to this work, the demonstration should be made for other Gram-positive species, including multi-drug resistant organisms. For instance *mecA* gene is also responsible for high level of resistance of methicillin-resistant *S. aureus* (MRSA) (Stapleton and Taylor, 2002; Li et al., 2012), but other types of resistance should also be explored.

The perspective and next challenges to this study would be: (i) to limit the number of time points and automate the sample handling; (ii) to expand the repertoire of drug/bacteria combinations, with additional types of antibiotic resistance other than betalactamase and *mecA*+ and in particular with multi-drug resistant organisms relevant for clinical practice such as *Staphylococcus aureus*; (iii) to show statistical significance by considering more biological replicates; (iv) to test the robustness of the technology by assessing performance with lower numbers of bacteria reflecting the range of bacterial concentrations observed in bacterially poor clinical samples; and (v) to test clinical samples such as urine, a generally good candidate for cytometry measurements and with a reasonable bacterial biomass in clinical cases. Achieving those objectives will probably require dedicated instrumental and protocol works to propose a research platform that is able to automate several steps of an optimized protocol and that would allow us to reach clinical statistical relevance for those various parameters.

## Data availability statement

The raw data supporting the conclusions of this article will be made available by the authors, without undue reservation.

## Author contributions

A-CC-K: choice and characterization of the biological model. SD and A-CC-K: design of the study and experiments. PM and ME: definition and implementation of the data analyses and analyses of the results. SD: analysis and article draft. SB: discussion insights. CK, CG, CV, QJ, FM, and FR: review of the results and draft improvement. FM, QJ, and FR: coordination of the project. All authors contributed to the article and approved the submitted version.

## Funding

This work was supported, in part, by the Agence Nationale de la Recherche through a grant awarded to BIOASTER (grant number ANR-10-AIRT-03) and by bioMérieux.

## Conflict of interest

The authors declare that the research was conducted in the absence of any commercial or financial relationships that could be construed as a potential conflict of interest.

## Publisher’s note

All claims expressed in this article are solely those of the authors and do not necessarily represent those of their affiliated organizations, or those of the publisher, the editors and the reviewers. Any product that may be evaluated in this article, or claim that may be made by its manufacturer, is not guaranteed or endorsed by the publisher.

## Figures and Tables

**Table:** 

ATCC	American type culture collection
AST	Antibiotic susceptibility testing
BSL1	Biosafety level 1 laboratory
c	Low breakpoint antibiotic concentration
C	High breakpoint antibiotic concentration
CLSI	Clinical and Laboratory Standards Institute
EC	*Escherichia coli*
FAD	(Fully oxidized) quinone form of Flavin Adenine Dinucleotide
FSC	Forward scattering
MHB	Mueller Hinton Broth
MIC	Minimum inhibitory concentration
NADH	Reduced form of Nicotinamide Adenine Dinucleotide
PBP	Penicillin-binding proteins
PCA	Principal components analysis
ROS	Reactive oxygen species
SE	*Staphylococcus epidermidis*
SSC	Side scattering
TCA	Tricarboxylic acid cycle

## Footnotes

^1^ https://CRAN.R-project.org/package=e1071
