# Supplementary material for: Single-cell scattering and auto-fluorescence-based fast antibiotic susceptibility testing for gram-negative and gram-positive bacteria
Source: Front Microbiol. 2023 Aug 4;14:1232250. doi: 10.3389/fmicb.2023.1232250 (PMC10436599; doi:10.3389/fmicb.2023.1232250)
Supplement: Supplementary file 1 [file Data_Sheet_1.pdf]

Supplementary Material

Single-cell scattering and auto-fluorescence-based fast antibiotic susceptibility testing for Gram-negative and Gram-positive bacteria

Sophie Dixneuf[1‡*], Anne-Coline Chareire-Kleiberg[2‡], Pierre Mahé[3], Meriem El Azami[5], Chloé Kolytcheff[5], Samuel Bellais[4], Cyril Guyard[1], Christophe Védrine[4], Frédéric Mallard[3], Quentin Josso[5], Fabian Rol[5]

*** Correspondence:** Corresponding Author: sophie.dixneuf@bioaster.org

1 Supplementary Tables

Table ST1. Comparison of broth microdillution experimental conditions for determining Minimal Inhibitory Concentrations according to CLSI standards and in the current study

	**Preculture**	**Medium of suspension**	**Turbidity (McF)**	**First dilution factor**	**Final concentration in microplate (McF and CFU/mL)**
**BMD (CLSI)**	Overnight COS agar plate (bioMérieux)	NaCl 0.85% (bioMérieux)	0.5	1/100 (50µL in 5mL of suspension medium)	0.0025McF ~7.5 10^5 CFU/mL (50µL in V_{well}=50µL of MHB)
**Current**	2h (EC) – 3h (SE) in liquid MHB	Mueller Hinton Broth (bioMérieux)	0.2	NA	0.1McF ~3. 10^7 CFU/mL (50µL in V_{well}=50µL of MHB)

Table ST2. Acquisition parameters on the LSRII flow cytometer

**Target**	**FSC**	**SSC**	**FAD**	**NADH**
**Excitation/Emisison maxima (nm)**			Exc. ~450 / Em. ~535	Exc. ~340 / Em. ~460
**Laser wavelength (nm)**	488	488	488	355
**Maximum laser output power (mW)**	100	100	100	50
**Collection anglae (°)**	2.29	90	90	90
**Long-pass cut-on wavelength (nm)**			505	
**Bandpass center / badwidth (nm)**	488 / 10	488 / 10	488 / 10	450 / 50
**PMT voltage**	574	241	688	574
**Measured parameters on each pulse (*i.e.* event)**	Area, Height	Area, Height	Area, Height	Area, Height

The threshold triggering the acquisition of an event was set at 200 on FSC-H (*i.e.* the height of the pulse measured in the FSC channel), and a Windows Extension of 4 was automatically applied, which generally guaranties that the entire area of the pulse can be measured in most cases. Neither gain, nor scaling factor was used when acquiring the data presented herein. As high sensitivity was required to capture autofluorescence compared to standard label-related fluorescence, PMTs voltages in the FAD and NADH channels were increased gradually until no further improvement in resolving fluorescence histograms of wild EC incubated for 2 hours with or without amoxicillin was observed anymore. No compensation was applied as no fluorescent labeling was used in this study. Sheath fluid speed was set at the "LO" position. While fine adjustment was constant within one single experiment, it was not necessarily reproducible from one experiment to another. As a result, one can only say that sheath fluid speed ranged between 6 and 24 µL/min, depending on the experiment. The number of events per second for a bacterial suspension at 0.1 McF ranged between 700 and 3500. The volume probed by sample was ~10µL.

Table ST3. Minimal Inhibitory Concentrations as measured according to CLSI standards and in the experimental conditions of the current cytometry study. Average of triplicates. End-point visual reading of the plate at 20 hour.

Strain	Phenotype	MIC (µg/mL)								
		Gentamicin			Amoxicillin			Oxacillin		
		Vitek®2	BMD	Current	Vitek®2	BMD	Current	Vitek®2	BMD	Current
EC1[*]	susceptible	<1	0.25 (0.25-1)[**]	0.5	NA	8	8	NA	NA	NA
EC2	resistant	>16	>64	>64	NA	>64	>64	NA	NA	NA
SE1	susceptible	<0.5	<0.03125	<0.03125	NA	NA	NA	<0.25	0.125	0.25
SE2	susceptible	<0.5	<0.03125	<0.03125	NA	NA	NA	<0.25	0.125	0.125
SE3	resistant	>16	16	>16	NA	NA	NA	>4	>16	>16
SE4	resistant	>16	>16	>16	NA	NA	NA	NA	>16	>16
*SA1**	susceptible				NA	NA	NA	NA	0.25 (0.12-0.5)[**]	0.25

[*] *Escherichia coli* EC1 (ATCC25922) and *Staphylococcus aureus* SA1 (ATCC29213) were used as quality control strains for the BMD method according to CLSI standards [CLSI_standard2015]

[**] MIC quality control ranges for the BMD method according to CLSI standards [CLSI_perf2017]

2 Supplementary Figures

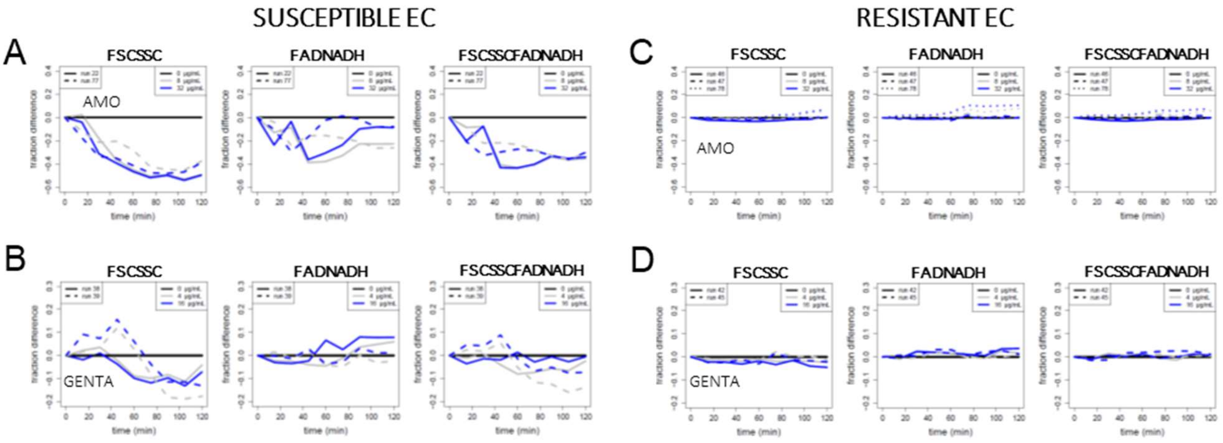

Figure S1. Illustration of inter-duplicate consistency of Susceptible/Resistant phenotyping for the *E. coli* model, based on the SVM analysis of the 2D scattering data (l-h-s panels), the 2D auto-fluorescence data (middle panels), and the 4D scattering-autofluorescence data (r-h-s panels), as explained Figure 2f-j. Antibiotic concentrations are 0 µg/mL (black curves), the low breakpoint c (grey curves) and the high breakpoint C (blue curves). Duplicates exp1 (full line) and exp2 (dotted line) for **(A)** EC1 (susceptible) + amoxicillin, **(B)** EC1 (susceptible) + gentamicin, **(C)** EC2 (resistant) + amoxicillin, and **(D)** EC2 (resistant) + gentamicin.

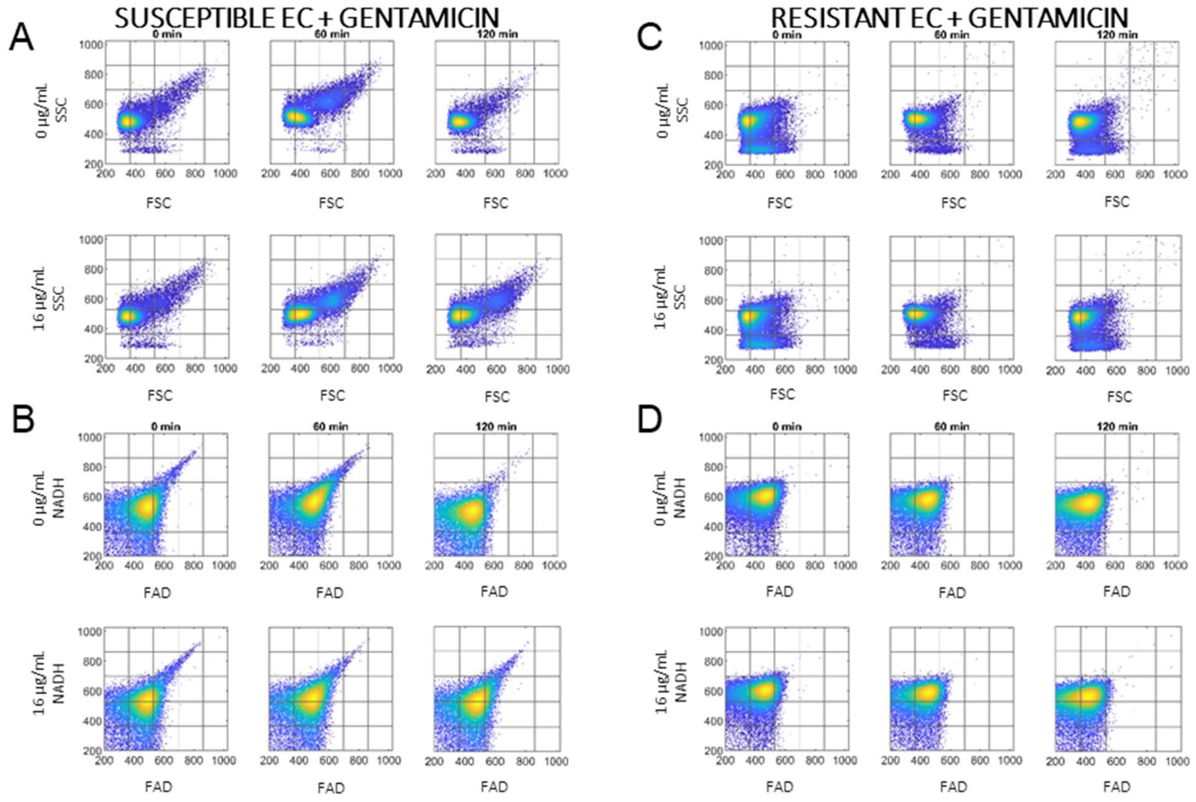

Figure S2. Example (one replicate) of the raw cytometry data for the EC + gentamicin model. **(A)** EC1 (susceptible), FSC-SSC space, **(B)** EC1 (susceptible), FAD-NADH space, **(C)** EC2 (resistant), FSC-SSC space, **(D)** EC2 (resistant), FAD-NADH space. 50000 events are shown on each 2D plot, and the lighter yellow color localizes the denser part of the distribution. For each strain and space combination, for the sake of readability, only two antibiotic concentrations are shown (0 µg/mL on the top row and C = 16 µg/mL on the bottom row), and three time points (0 min on the l-h-s column, 60 min in the middle column, and 120 min on the r-h-s column).

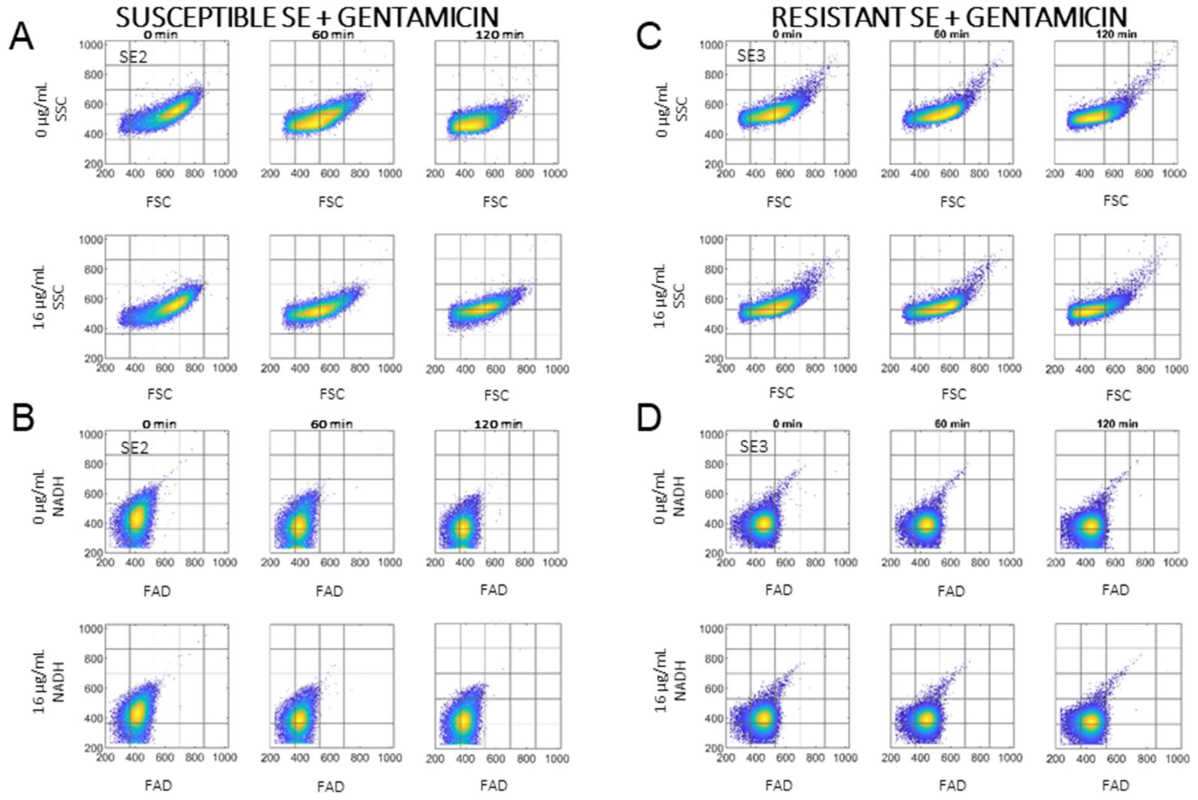

Figure S3. Example (one replicate) of the raw cytometry data for the SE + gentamicin model. **(A)** SE2 (susceptible), FSC-SSC space, **(B)** SE2 (susceptible), FAD-NADH space, **(C)** SE3 (resistant), FSC-SSC space, **(D)** SE3 (resistant), FAD-NADH space. 50000 events are shown on each 2D plot, and the lighter yellow color localizes the denser part of the distribution. For each strain and space combination, for the sake of readability, only two antibiotic concentrations are shown (0 µg/mL on the top row and C = 16 µg/mL on the bottom row), and three time points (0 min on the l-h-s column, 60 min in the middle column, and 120 min on the r-h-s column).

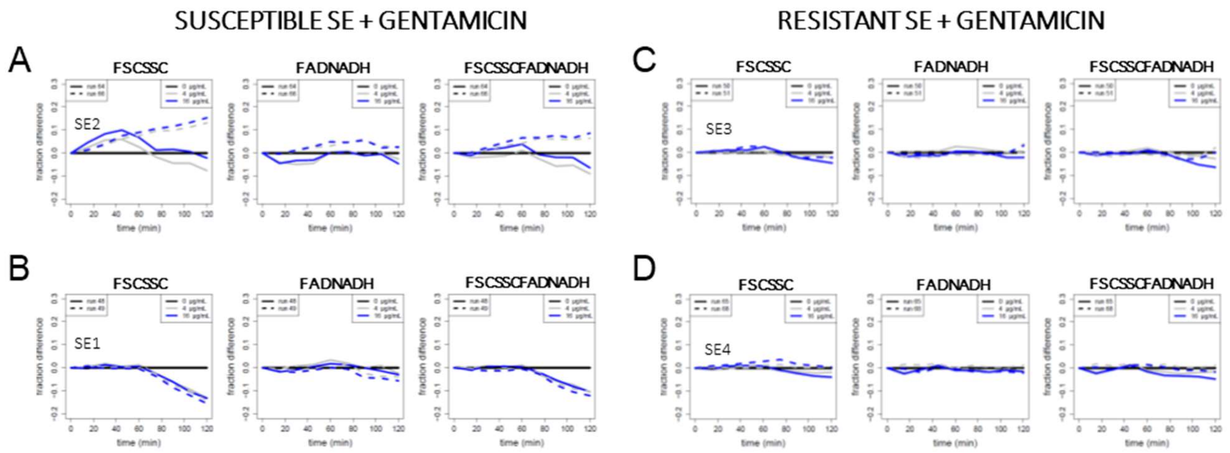

Figure S4. Illustration of inter-duplicate and inter-strain consistency of Susceptible/Resistant phenotyping for the *S. epidermidis* + gentamicin model, based on the SVM analysis of the 2D scattering data (l-h-s panels), the 2D auto-fluorescence data (middle panels), and the 4D scattering-autofluorescence data (r-h-s panels), as explained Figure 2f-j. Antibiotic concentrations are 0 µg/mL (black curves), the low breakpoint c=4µg/mL (grey curves) and the high breakpoint C=16µg/mL (blue curves). Duplicates exp1 (full line) and exp2 (dotted line) for **(A)** SE2 (susceptible), **(B)** SE1 (susceptible), **(C)** SE3 (resistant), and **(D)** SE4 (resistant).

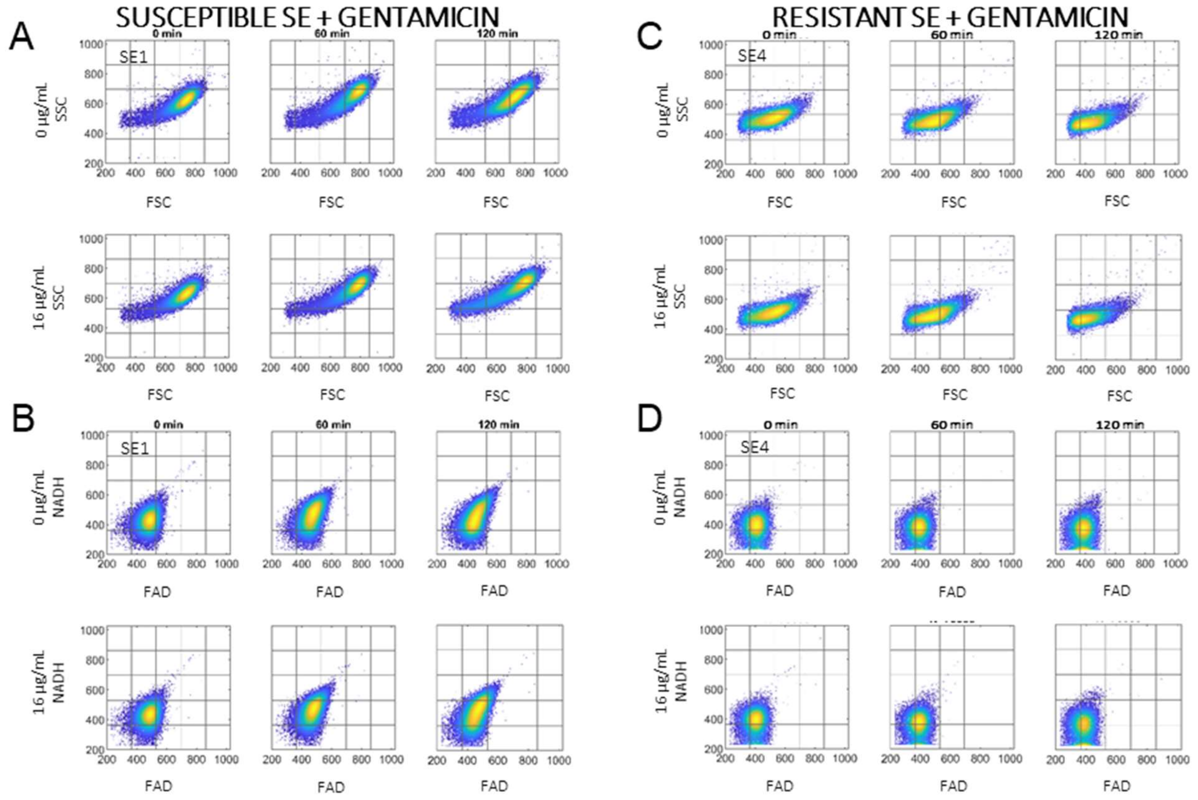

Figure S5. Example (one replicate) of the raw cytometry data for the SE + gentamicin model. **(A)** SE1 (susceptible), FSC-SSC space, **(B)** SE1 (susceptible), FAD-NADH space, **(C)** SE4 (resistant), FSC-SSC space, **(D)** SE4 (resistant), FAD-NADH space. 50000 events are shown on each 2D plot, and the lighter yellow color localizes the denser part of the distribution. For each strain and space combination, for the sake of readability, only two antibiotic concentrations are shown (0 µg/mL on the top row and C = 16 µg/mL on the bottom row), and three time points (0 min on the l-h-s column, 60 min in the middle column, and 120 min on the r-h-s column).

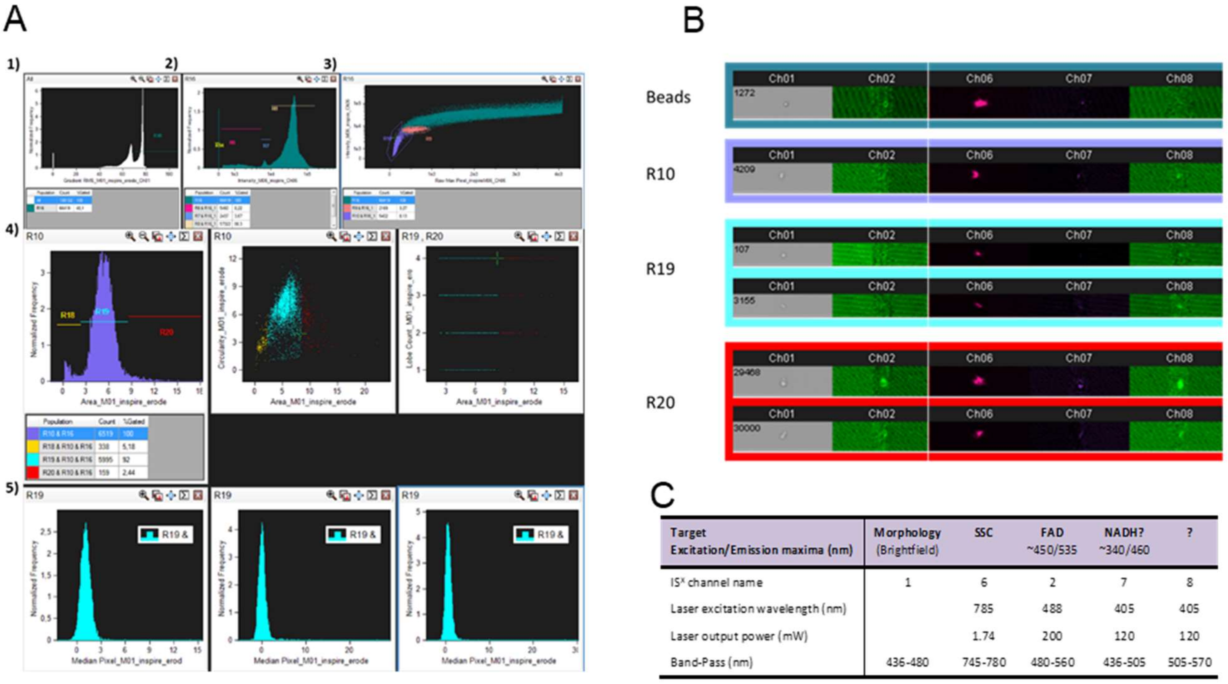

C

Target<br>Excitation/Emission maxima (nm)	Morphology<br>(Brightfield)	SSC	FAD<br>~450/535	NADH?<br>~340/460	?
IS^X channel name	1	6	2	7	8
Laser excitation wavelength (nm)		785	488	405	405
Laser output power (mW)		1.74	200	120	120
Band-Pass (nm)	436-480	745-780	480-560	436-505	505-570

Figure S6. Analysis of the incubation SE2 + gentamicin with the ImageStream-X instrument (**experiment *Ctrl1***). **A**) Gating pipeline; **B**) samples of images in the different gates for the different channels measured; **C**) Instrumental settings in each channel.

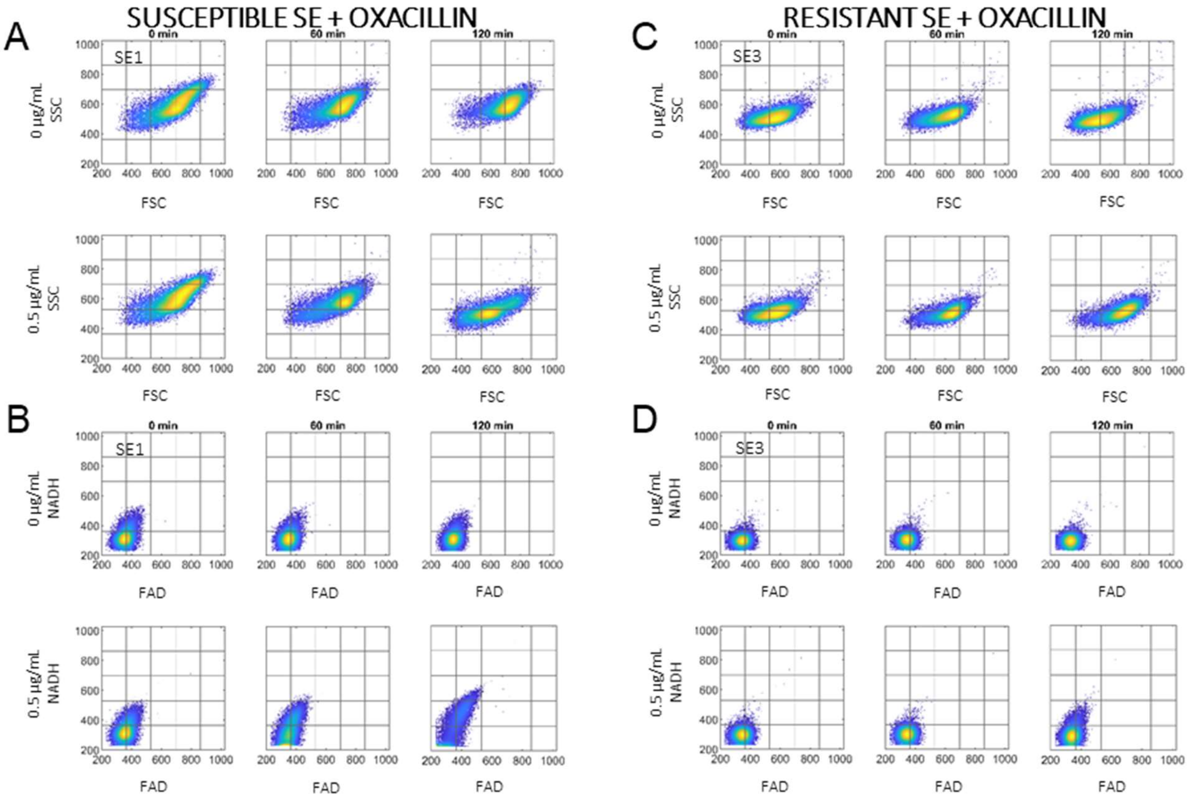

Figure S7. Example (one replicate) of the raw cytometry data for the SE + oxacillin model. **(A)** SE1 (susceptible), FSC-SSC space, **(B)** SE1 (susceptible), FAD-NADH space, **(C)** SE3 (*mecA*+), FSC-SSC space, **(D)** SE3 (*mecA*+), FAD-NADH space. 50000 events are shown on each 2D plot, and the lighter yellow color localizes the denser part of the distribution. For each strain and space combination, for the sake of readability, only two antibiotic concentrations are shown (0 µg/mL on the top row and C = 0.5 µg/mL on the bottom row), and three time points (0 min on the l-h-s column, 60 min in the middle column, and 120 min on the r-h-s column).

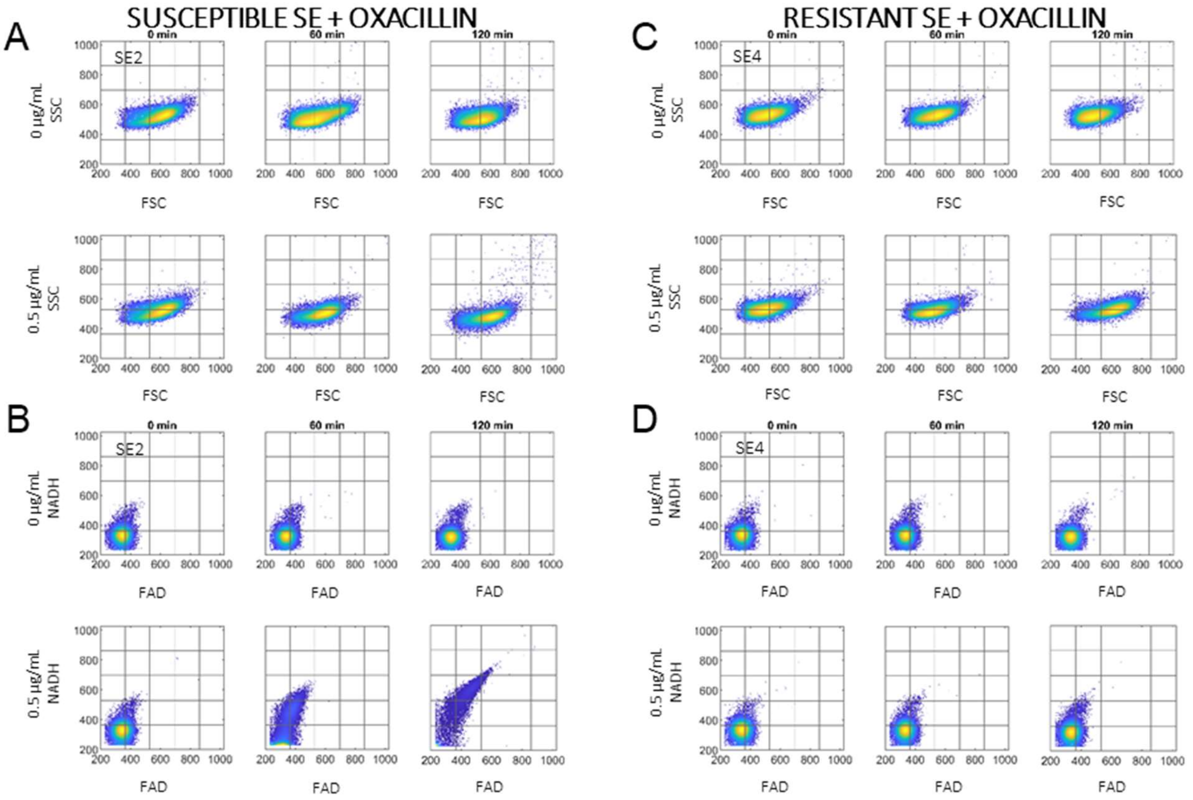

Figure S8. Example (one replicate) of the raw cytometry data for the SE + oxacillin model. **(A)** SE2 (susceptible), FSC-SSC space, **(B)** SE2 (susceptible), FAD-NADH space, **(C)** SE4 (*mecA*+), FSC-SSC space, **(D)** SE4 (*mecA*+), FAD-NADH space. 50000 events are shown on each 2D plot, and the lighter yellow color localizes the denser part of the distribution. For each strain and space combination, for the sake of readability, only two antibiotic concentrations are shown (0 µg/mL on the top row and C = 0.5 µg/mL on the bottom row), and three time points (0 min on the l-h-s column, 60 min in the middle column, and 120 min on the r-h-s column).

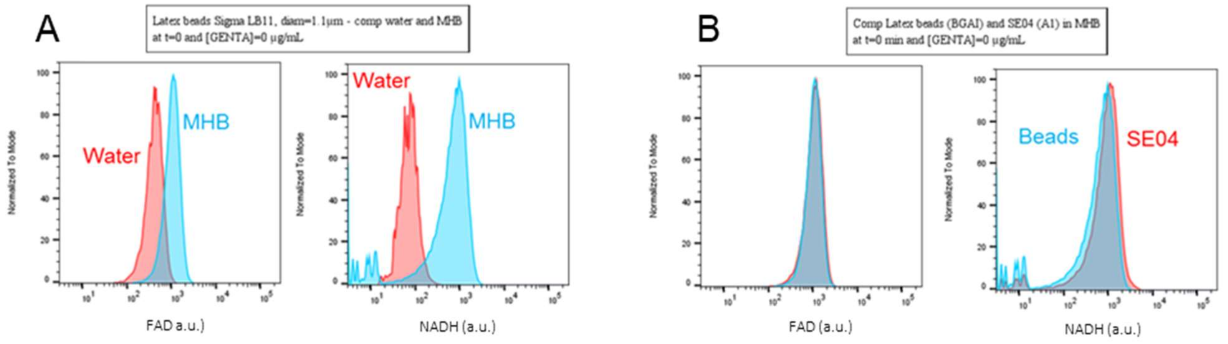

Figure S9. Assessment of the environmental fluorescence and its impact on the bacterial intracellular auto-fluorescence detection, as measured with the LSRII flow cytometer in the FAD and NADH channels (**experiment *Ctrl2***). **(A)** Non-stained polystyrene beads (Sigma-Aldrich, ref. LB11, ϕ=1.1µm) in MHB (blue) or in water (red). **(B)** Non-stained polystyrene beads (blue) and SE4 strain (red), both in MHB.

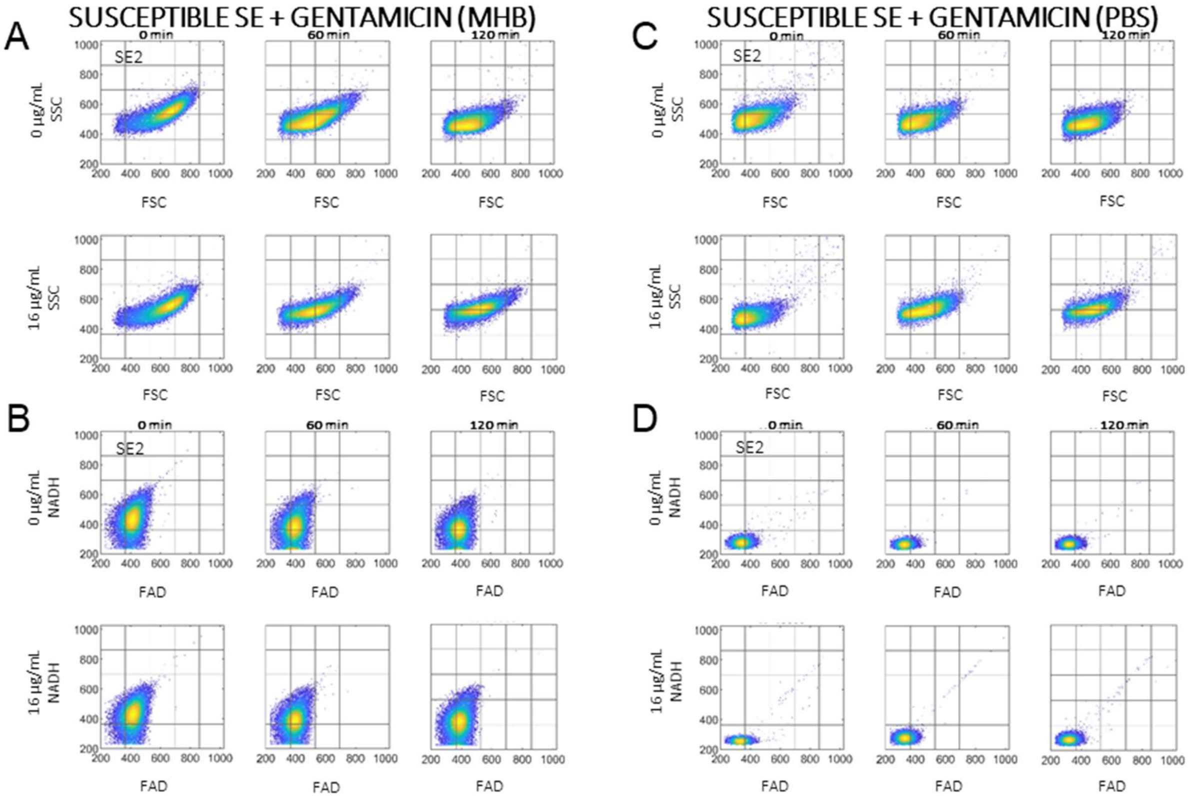

Figure S10. Comparison of the raw cytometry data for the incubation of SE2 (susceptible) + gentamicin, measured either **(A-B)** directly in MHB (current protocol) or **(C-D)** after washing in PBS (**experiment *Ctrl3***). **(A,C)** FSC-SSC space, **(B,D)** FAD-NADH space. For the sake of readability, only two antibiotic concentrations are shown (0 µg/mL on the top row and C = 16 µg/mL on the bottom row), and three time points (0 min on the l-h-s column, 60 min in the middle column, and 120 min on the r-h-s column).

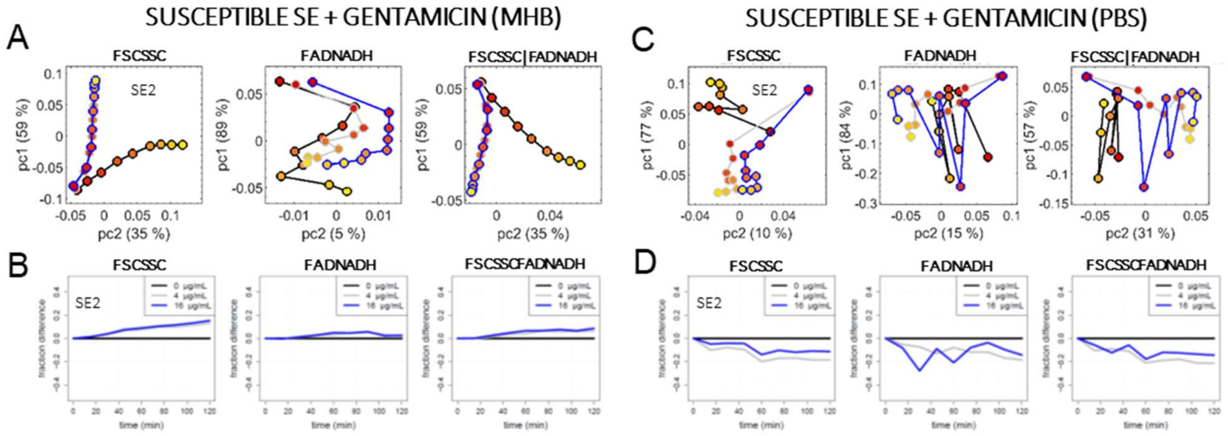

Figure S11. PCA and OC-SVM analysis of the raw data shown in Figure S9. Comparison of the incubation of SE2 (susceptible) + gentamicin, measured either **(A-B)** directly in MHB (current protocol) or **(C-D)** after washing in PBS (**experiment *Ctrl3***). **(A,C)** pc2 versus pc1 scores, for the 2D scattering data (l-h-s), the 2D auto-fluorescence data (center), and the concatenation of the 2D scattering and the 2D auto-fluorescence data (r-h-s). (**B,D**) Fraction of events remaining in the reference (t=0 min) SVM support over time, for the 2D scattering data (l-h-s), the 2D auto-fluorescence data (center), and the 4D scattering-auto-fluorescence data (r-h-s).

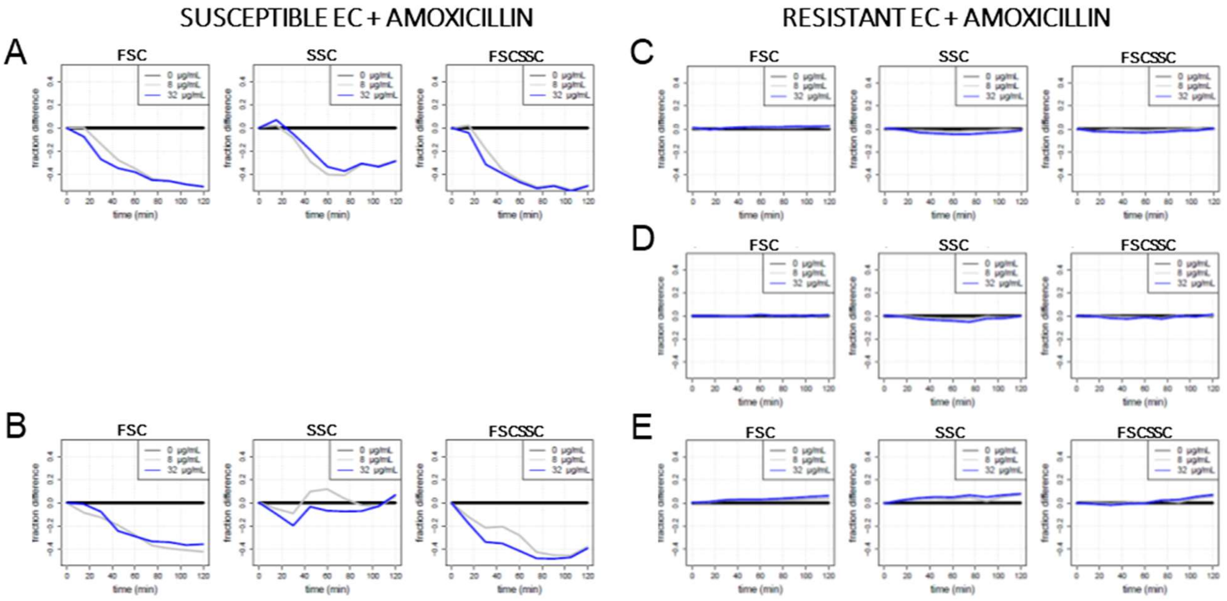

Figure S12. OC-SVM analysis of the scattering data for the case of EC incubated with amoxicillin, in either 1D or 2D spaces: FSC (l-h-s panels), SSC (middle panels), and 2D FSC-SSC (r-h-s panels). Antibiotic concentrations are 0 μg/mL (black curves), the low breakpoint c=4μg/mL (grey curves) and the high breakpoint C=16μg/mL (blue curves). **(A-B)** EC1 (susceptible), **(C-E)** EC2 (resistant).

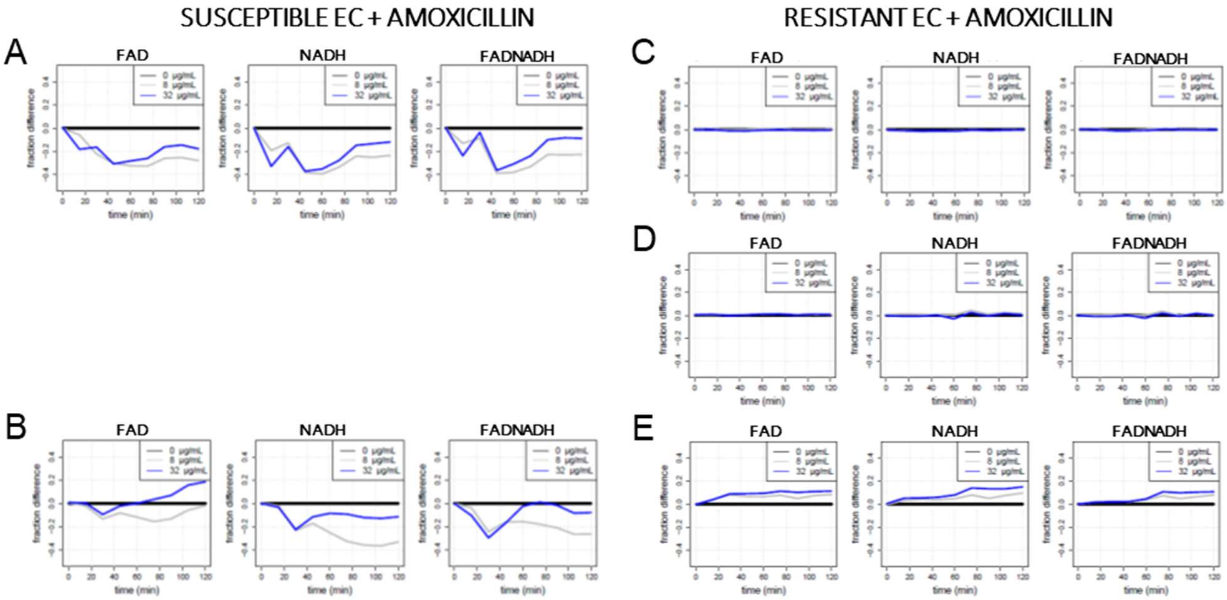

Figure S13. OC-SVM analysis of the autofluorescence data for the case of EC incubated with amoxicillin, in either 1D or 2D spaces: FAD (l-h-s panels), NADH (middle panels), and 2D FAD-NADH (r-h-s panels). Antibiotic concentrations are 0 μg/mL (black curves), the low breakpoint c=4μg/mL (grey curves) and the high breakpoint C=16μg/mL (blue curves). **(A-B)** EC1 (susceptible), **(C-E)** EC2 (resistant).

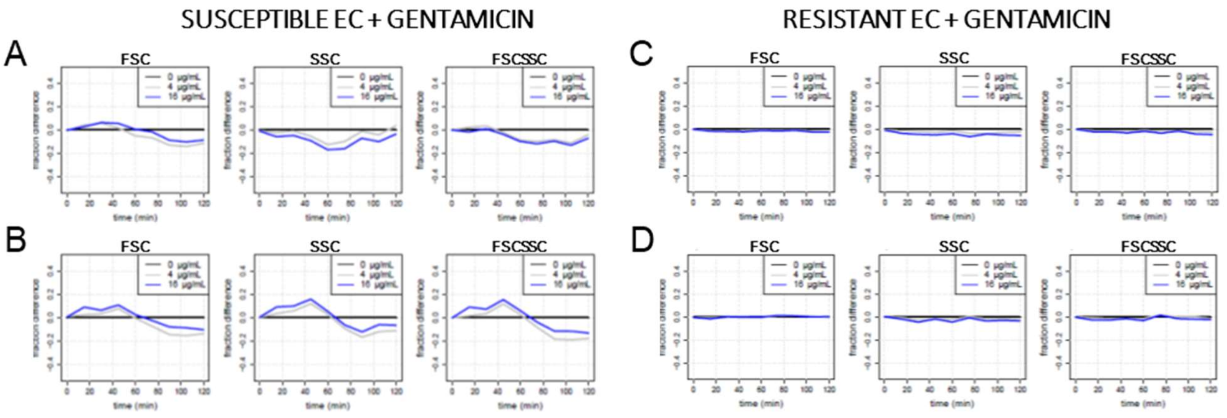

Figure S14. OC-SVM analysis of the scattering data for the case of EC incubated with gentamicin, in either 1D or 2D spaces: FSC (l-h-s panels), SSC (middle panels), and 2D FSC-SSC (r-h-s panels). Antibiotic concentrations are 0 µg/mL (black curves), the low breakpoint c=4µg/mL (grey curves) and the high breakpoint C=16µg/mL (blue curves). **(A-B)** EC1 (susceptible), **(C-D)** EC2 (resistant).

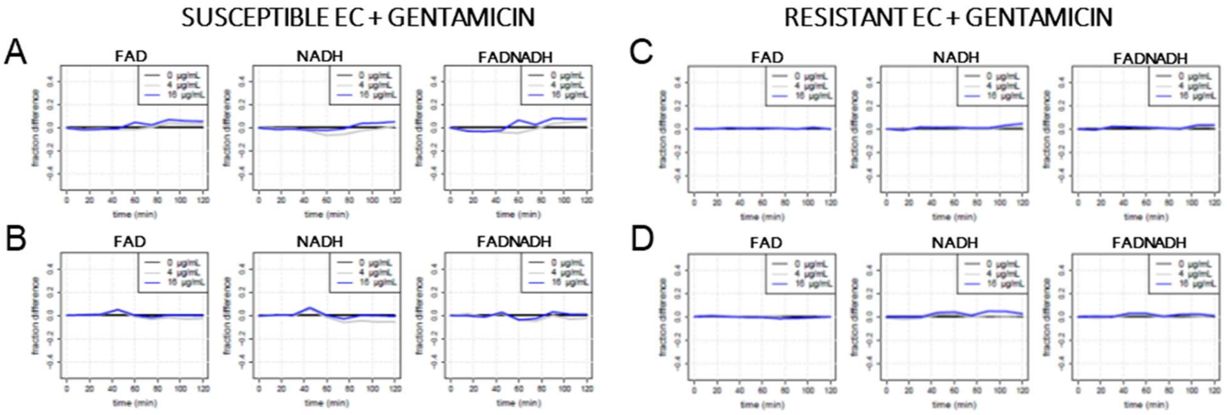

Figure S15. OC-SVM analysis of the autofluorescence data for the case of EC incubated with gentamicin, in either 1D or 2D spaces: FAD (l-h-s panels), NADH (middle panels), and 2D FAD-NADH (r-h-s panels). Antibiotic concentrations are 0 µg/mL (black curves), the low breakpoint c=4µg/mL (grey curves) and the high breakpoint C=16µg/mL (blue curves). **(A-B)** EC1 (susceptible), **(C-D)** EC2 (resistant).

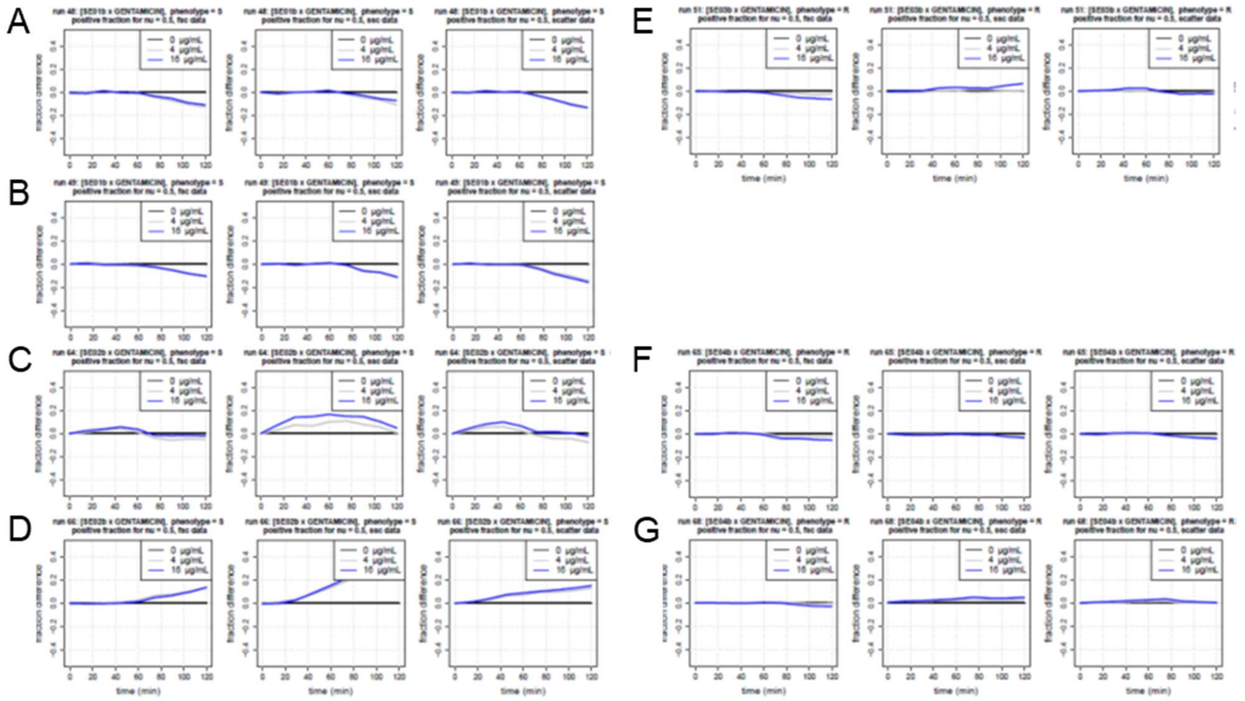

Figure S16. OC-SVM analysis of the scattering data for the case of SE incubated with gentamicin, in either 1D or 2D spaces: FSC (l-h-s panels), SSC (middle panels), and 2D FSC-SSC (r-h-s panels). Antibiotic concentrations are 0 µg/mL (black curves), the low breakpoint c=4µg/mL (grey curves) and the high breakpoint C=16µg/mL (blue curves). **(A-B)** SE1 (susceptible), **(C-D)** SE2 (susceptible), **(E)** SE3 (resistant), **(F-G)** SE4 (resistant). (*Note*: FITC stands for FAD and QDOT stands for NADH)

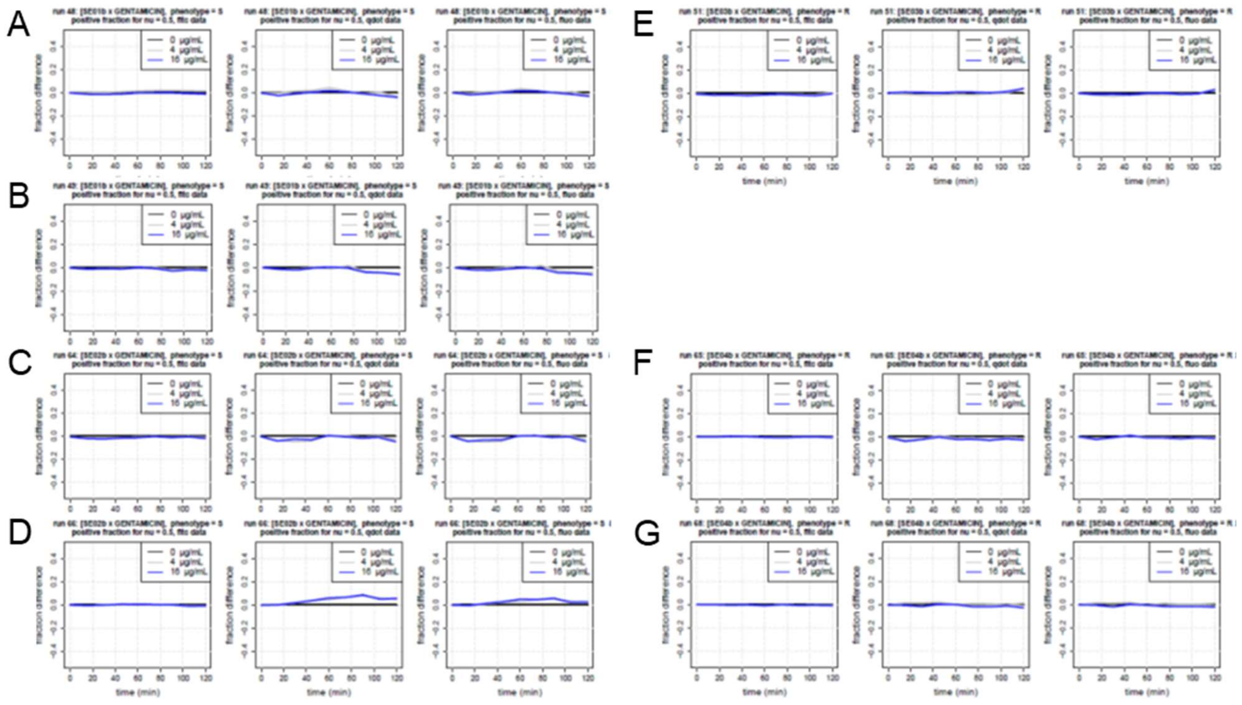

Figure S17. OC-SVM analysis of the autofluorescence data for the case of SE incubated with gentamicin, in either 1D or 2D spaces: FAD (l-h-s panels), NADH (middle panels), and 2D FAD-NADH (r-h-s panels). Antibiotic concentrations are 0 µg/mL (black curves), the low breakpoint c=4µg/mL (grey curves) and the high breakpoint C=16µg/mL (blue curves). **(A-B)** SE1 (susceptible), **(C-D)** SE2 (susceptible), **(E)** SE3 (resistant), **(F-G)** SE4 (resistant). (*Note*: FITC stands for FAD and QDOT stands for NADH)

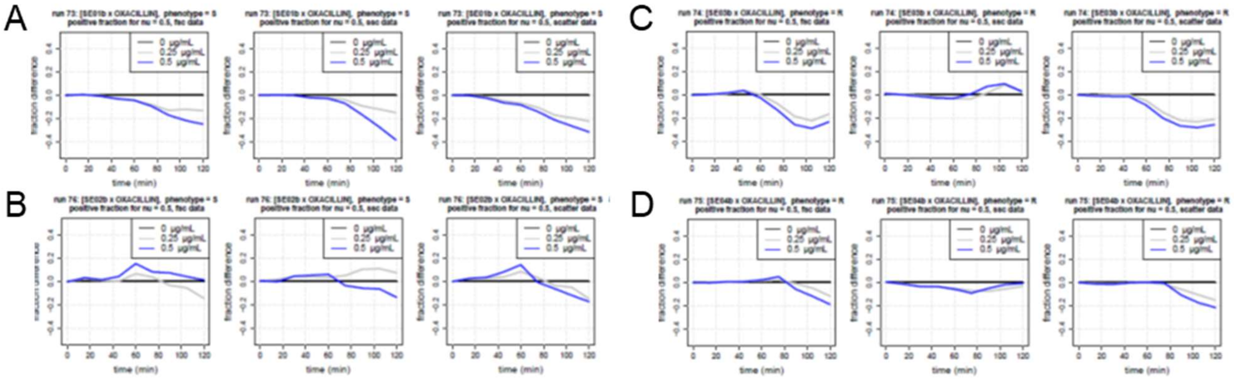

Figure S18. OC-SVM analysis of the scattering data for the case of SE incubated with oxacillin, in either 1D or 2D spaces: FSC (l-h-s panels), SSC (middle panels), and 2D FSC-SSC (r-h-s panels). Antibiotic concentrations are 0 µg/mL (black curves), the low breakpoint c=4µg/mL (grey curves) and the high breakpoint C=16µg/mL (blue curves). **(A)** SE1 (susceptible), **(B)** SE2 (susceptible), **(C)** SE3 (resistant), **(D)** SE4 (resistant). (*Note*: FITC stands for FAD and QDOT stands for NADH)

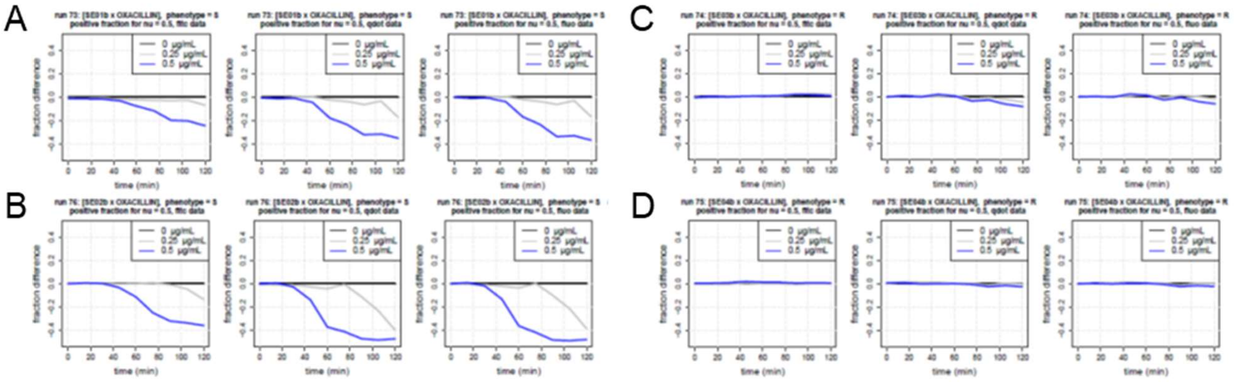

Figure S19. OC-SVM analysis of the autofluorescence data for the case of SE incubated with oxacillin, in either 1D or 2D spaces: FAD (l-h-s panels), NADH (middle panels), and 2D FAD-NADH (r-h-s panels). Antibiotic concentrations are 0 µg/mL (black curves), the low breakpoint c=4µg/mL (grey curves) and the high breakpoint C=16µg/mL (blue curves). **(A)** SE1 (susceptible), **(B)** SE2 (susceptible), **(C)** SE3 (resistant), **(D)** SE4 (resistant). (*Note*: FITC stands for FAD and QDOT stands for NADH)
